# Rational design of indoleamine 2,3-dioxygenase 1 (IDO1) inhibitors featuring 1,2,3-triazole derivatives with enhanced anti-inflammatory and analgesic efficacy

**DOI:** 10.3389/fphar.2025.1574007

**Published:** 2025-09-19

**Authors:** Qingying Liu, Xixi Hou, Yueliang Wang, Mingyue Tian, Baoyu He, Jingjing Guo, Jianxue Yang

**Affiliations:** ^1^ The First Affiliated Hospital, and College of Clinical Medicine of Henan University of Science and Technology, Luoyang, China; ^2^ Department of Pain Management, the First Affiliated Hospital, Zhengzhou University, Zhengzhou, China; ^3^ Centre for Artificial Intelligence Driven Drug Discovery, Faculty of Applied Sciences, Macao Polytechnic University, Macau, China

**Keywords:** 1,2,3-triazoles, IDO1 inhibitor, 2H-benzo[b][1,4]oxazin-3(4H)-one, anti-inflammatory, analgesic

## Abstract

This study applied a target-based drug design approach focused on the IDO1 enzyme, which features a heme active site. By introducing a 1,2,3-triazole moiety capable of coordinating with the ferrous ion in heme, a series of 2H-benzo[b][1,4]oxazin-3(4H)-one derivatives were designed. Enzyme assays demonstrated that these compounds generally inhibited IDO1 activity, with Compound **14e** showing the most potent effect, achieving an IC_50_ value of 3.63 μM. Molecular docking studies indicated that the 1,2,3-triazole ring in Compound **14e** is positioned directly above the heme, forming a coordination bond with the ferrous ion. Additionally, it engages in π-π interactions with Phe263, while the amide group of the 2H-benzo[b][1,4]oxazin-3(4H)-one scaffold forms hydrogen bonds with Lys238. *In vivo* experiments in mice showed that Compound **14e** significantly reduced CFA-induced upregulation of Iba1 in the spinal dorsal horn and alleviated mechanical hypersensitivity, thermal hyperalgesia, and spontaneous pain. Moreover, treatment with Compound **14e** led to a significant reduction in the levels of pro-inflammatory cytokines TNF-α and IL-1β in CFA-treated mice. Importantly, Compound **14e** demonstrated a favorable safety profile, with no observed toxicity in major organs, highlighting its potential as a promising anti-inflammatory and analgesic agent targeting IDO1.

## 1 Introduction

Pain is an unpleasant sensory and emotional experience resulting from actual or potential tissue damage, serving as a protective response to harmful stimuli. While acute pain acts as an alert mechanism within the body and is typically manageable with conventional analgesics, chronic pain poses a more significant challenge. It is often associated with complications such as poor sleep, anxiety, and depression, which can, in turn, exacerbate the pain. Furthermore, chronic pain may be accompanied by comorbid conditions that increase the overall burden on the patient, reduce the quality of life, and, in severe cases, pose life-threatening risks. Currently, more than 30% of the global population suffers from chronic pain. Although pharmacotherapy remains the primary treatment approach, many existing analgesics have notable limitations, including the development of tolerance, risk of dependence, hepatotoxicity, and insufficient efficacy. Thus, there is an urgent need for the development of novel analgesic drugs to overcome these challenges.

Recent studies have identified a strong link between pain and tryptophan metabolism, particularly the tryptophan-kynurenine pathway. L-tryptophan is metabolized via this pathway to produce neurotoxic metabolites, such as 3-hydroxykynurenine (3-HK) and quinolinic acid (QUIN), which can induce neuronal damage and neuroinflammation, contributing to neuropathic pain. The accumulation of these metabolites perpetuates neuroinflammation, further exacerbating chronic pain (Rojewska et al., 2018). Moreover, increased metabolism of L-tryptophan through the kynurenine pathway reduces its conversion to serotonin. Low serotonin levels are associated with mood disorders, including depression and anxiety, which frequently co-occur with chronic pain, creating a detrimental cycle (Munawar et al., 2023). Therefore, targeting the tryptophan-kynurenine pathway presents a promising therapeutic strategy for pain management. Indoleamine 2,3-dioxygenase 1 (IDO1) is a key rate-limiting enzyme in the tryptophan-kynurenine pathway, catalyzing the breakdown of L-tryptophan (Serafini et al., 2020). Under normal physiological conditions, IDO1 is expressed at low levels, but its expression can be significantly upregulated by pro-inflammatory cytokines such as IL-1β and TNF-α, as well as by immune activation (Gao et al., 2025). This overexpression disrupts the balance of tryptophan metabolism, leading to excessive conversion of L-tryptophan to kynurenine and reducing its conversion to serotonin. Consequently, inhibiting IDO1 expression may enhance serotonin synthesis and provide neuroprotection. Studies have shown that IDO1 plays a critical role in neuropathic pain, and the use of IDO1 inhibitors has been demonstrated to alleviate pain in animal models (Wang et al., 2020). Moreover, genetic knockout of the IDO1 gene has been found to reduce pain-related behaviors in mice, highlighting IDO1 as a potential target for the development of novel analgesic drugs (Kim et al., 2012).

**FIGURE 1:**
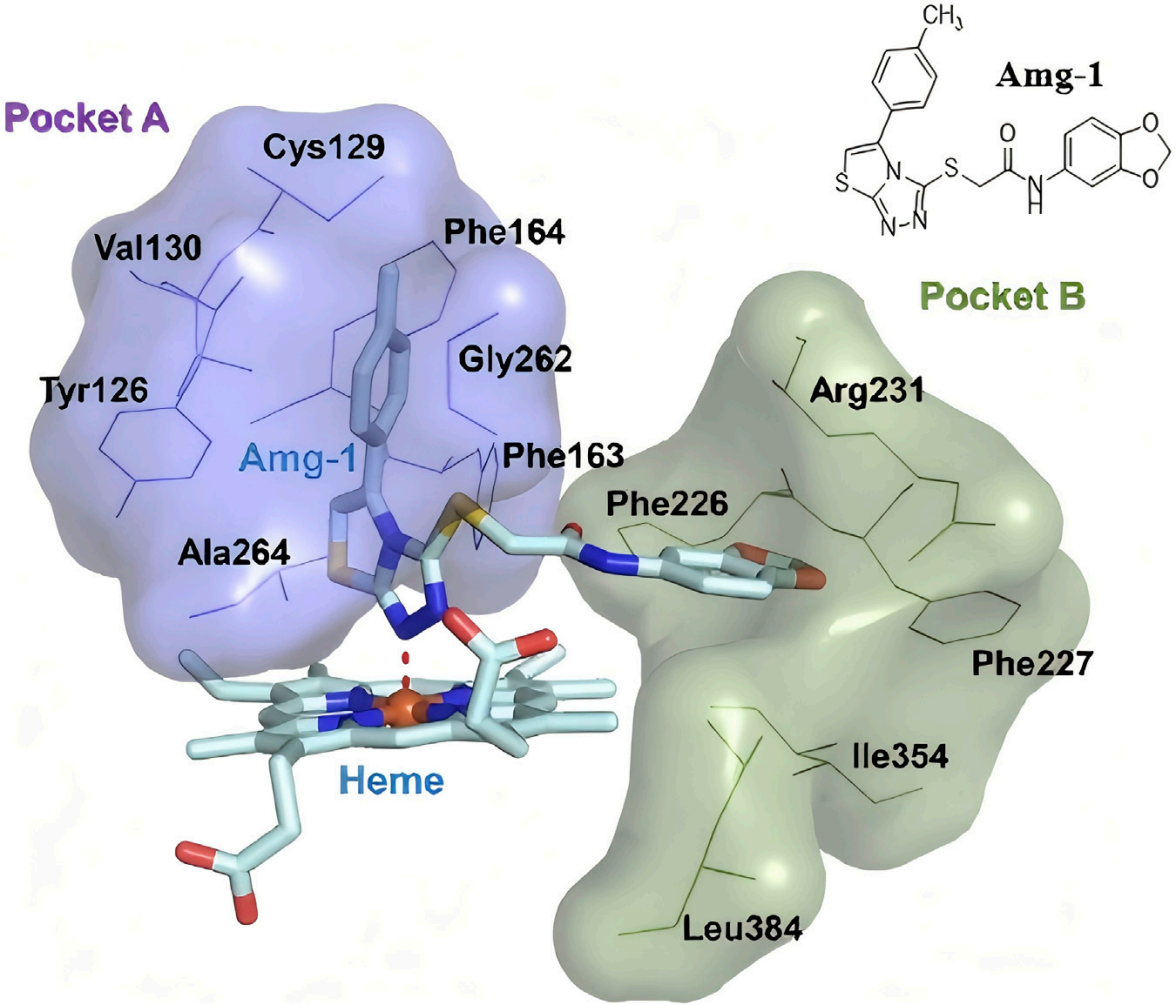
The crystal structure of the IDO1 protein in complex with the small ligand molecule Amg-1 (4PK5).

IDO1 is a heme-containing monomeric enzyme. Structural analysis of the IDO1-ligand complex with the small molecule Amg-1 (PDB: 4PK5) reveals that the heme group serves as the primary active site of the enzyme (Hou et al., 2022; Röhrig et al., 2012; Tojo et al., 2014). The enzyme features two large active pockets, one of which is capable of accommodating larger ligands (Figure 1). Pocket A is hydrophobic, and the 1,2,4-triazole moiety of Amg-1 coordinates with the heme iron, while the 4-methylphenyl ring penetrates deeply into Pocket A, forming hydrophobic interactions with surrounding amino acids (Mao et al., 2020; Xu et al., 2022). Therefore, designing small molecules that can enter IDO1, coordinate with the heme group, and form hydrophobic interactions with Pocket A, as well as interact with residues in Pocket B, may effectively inhibit IDO1 activity and disrupt its physiological function.

**FIGURE 2:**
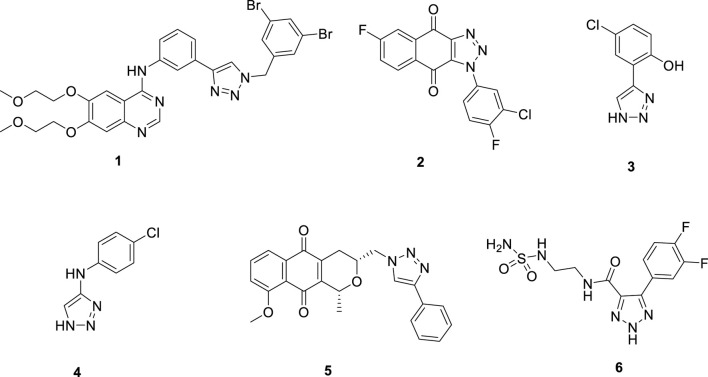
1,2,3-triazole compounds as potential IDO1 inhibitors.

The 1,2,3-triazole ring is a highly stable five-membered aromatic heterocycle containing three nitrogen atoms, which provide multiple coordination sites and confer mild acidity. This structure enables strong metal coordination. Additionally, the π-π conjugation and large dipole moment of the 1,2,3-triazole facilitate non-covalent interactions, such as hydrophobic effects, van der Waals forces, and hydrogen bonding, with various biological receptors. The 1,2,3-triazole motif exhibits diverse pharmacological activities, including anti-tumor, antiviral, anti-inflammatory, and antibacterial effects, making it a valuable scaffold for the design of IDO1 inhibitors (Augsten et al., 2023; Guan et al., 2024; Pereira et al., 2021). For example, Hou’s research group utilized the terminal alkyne of the EGFR inhibitor Erlotinib to synthesize a series of 1,2,3-triazole derivatives through click chemistry. Among these, compound **1** (Figure 2) demonstrated significant IDO1 inhibition (IC_50_ = 0.59 μM), and molecular docking studies indicated that the 1,2,3-triazole ring coordinates with the heme group of IDO1 (Hou et al., 2022). Similarly, Yang’s team designed and synthesized 1-phenyl-1H-naphtho [2,3-d][1,2,3]triazole-4,9-dione derivatives. Compound **2** (Figure 2) exhibited potent IDO1 inhibition (IC_50_ = 5 nM) and demonstrated significant anti-tumor activity in vivo models of Lewis lung carcinoma (LL2) and hepatocellular carcinoma (Hepa1-6), with minimal toxicity (Pan et al., 2020). Zoete’s group applied computational modeling to develop an IDO1 inhibitor based on a 4-phenyl-1,2,3-triazole scaffold. Their compound **3** (Figure 2), showed an IC_50_ of 330 nM in enzymatic assays and no activity against TDO, indicating high selectivity. In cellular assays, compound **3** displayed IC_50_ values of 2 nM for mouse IDO1 and 80 nM for human IDO1, with minimal cytotoxicity (Rohrig et al., 2012; Rohrig et al., 2019). Compound **4** (Figure 2) was identified as a reversible, non-competitive IDO1 inhibitor with IC_50_ values of 11.3 μM in enzymatic assays and 0.023 μM in cellular assays. Its strong cellular activity and binding affinity for IDO1 were further supported by biochemical, spectroscopic, and crystallographic analyses, which revealed that the formation of a stable ferrous IDO1–4 complex, with slow association and dissociation kinetics, plays a key role in its potency (Alexandre et al., 2018). Additionally, the pyranonaphthoquinone derivative **5** (Figure 2) demonstrated micromolar-level potency against IDO1 (IC_50_ = 6 μM) with minimal cytotoxicity. It significantly reduced kynurenine production by over 60% in treated cells at low concentrations (Wang et al., 2019). Manna’s research group developed a series of 4,5-disubstituted 1,2,3-triazoles, with compound **6** (Figure 2) showing strong IDO1 inhibition in MDA-MB-231 cells and negligible cytotoxicity. T cell activity studies indicated that these potent inhibitors could effectively regulate IDO1 activity, inducing an immune response against breast cancer cells (Panda et al., 2019). In summary, the 1,2,3-triazole structure has proven to be a valuable scaffold for developing novel IDO1 inhibitors, offering significant potential for treating diseases associated with aberrant tryptophan metabolism.

**FIGURE 3:**
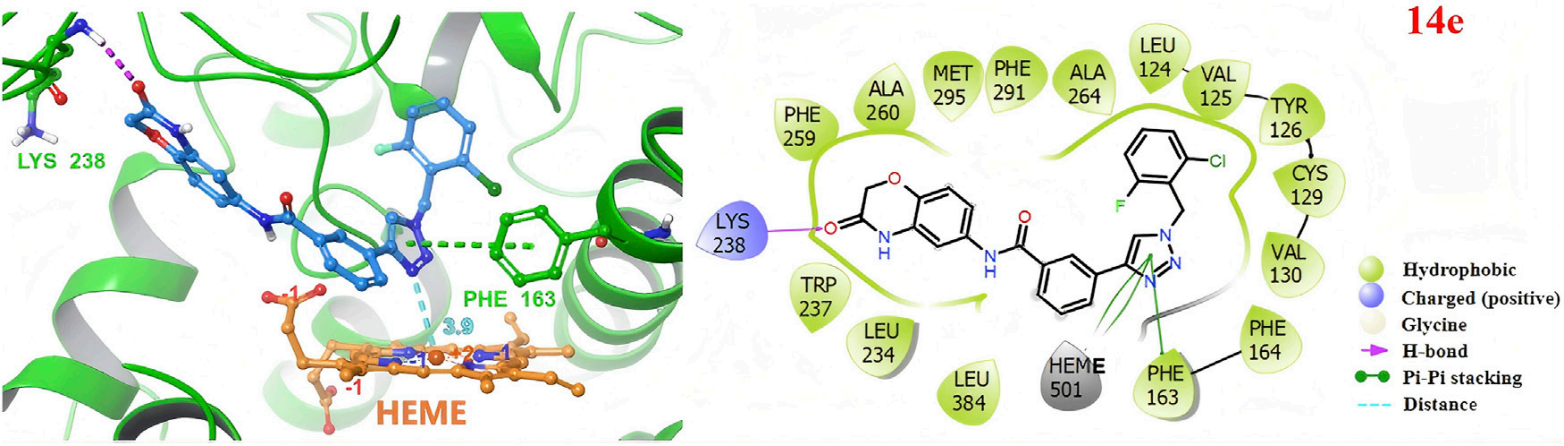
Predicted 3D and 2D binding modes of compound **14e** with indoleamine 2,3-dioxygenase 1 (IDO1). The overall structure of human IDO1 is illustrated in a cartoon representation (PDB ID: 4PK5). Compounds **14e** and heme (orange) are depicted using stick models. Red and yellow dashed lines indicate hydrogen bonds, while pi-pi stacking are depicted as green dashed lines.

**SCHEME 1:**
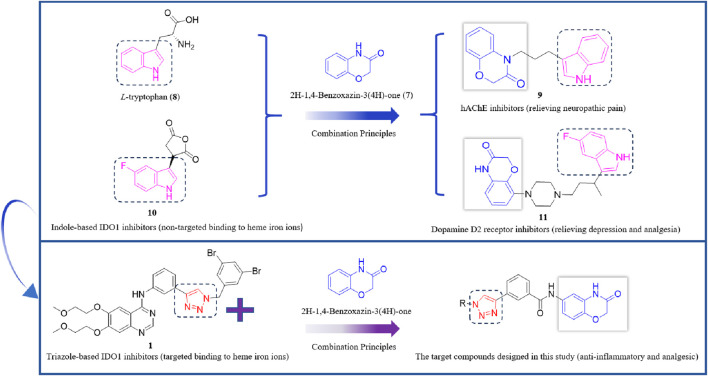
Design route of IDO1 inhibitors with a 1,2,3-triazole structure.

2H-1,4-Benzoxazin-3(4H)-one (**7**) is a significant nitrogen- and oxygen-containing heterocyclic compound, renowned for its broad biological activities and low toxicity, making it a promising scaffold for developing various analgesic drug candidates (Figure 3). Molecular docking studies have examined the incorporation of the indole core structure of L-tryptophan (**8**) at the 4-position nitrogen atom of the 2H-1,4-Benzoxazin-3(4H)-one framework, resulting in compound **9**, which functions as a hAChE inhibitor and alleviates nerve-related pain. Additionally, the 5-fluoroindole core structure of the IDO1 inhibitor compound 10 has been integrated at the 8-position carbon atom of the 2H-1,4-Benzoxazin-3(4H)-one scaffold, leading to compound **11**. This compound exhibits potent dopamine D2 receptor inhibition and high activity in inhibiting 5-HT reuptake, which significantly increases serotonin levels in the synaptic cleft, showing potential for alleviating depression and providing analgesic effects (Crosignani et al., 2017; González-Gutiérrez et al., 2025; Mendez-Rojas et al., 2018). Indole-based IDO1 inhibitors, such as compound **10**, do not directly interact with the ferrous ion in the heme of IDO1. Instead, they primarily exert their inhibitory effects through hydrogen bonding between the amine group and Ser167. As a result, their inhibitory potency is weaker compared to small molecules that directly interact with the ferrous ion in the heme center. Building on this, we hypothesize that incorporating a 1,2,3-triazole group—capable of coordinating with the ferrous ion in the heme—into the 2H-1,4-Benzoxazin-3(4H)-one structure could yield novel lead compounds for IDO1 inhibition. Given that many reported IDO1 inhibitors, such as compound **1**, feature halogenated aromatic rings, we plan to introduce a halogenated benzyl group onto the 1,2,3-triazole structure, advancing the development of potential analgesic drugs (Scheme 1).

## 2 Chemistry

**SCHEME 2:**
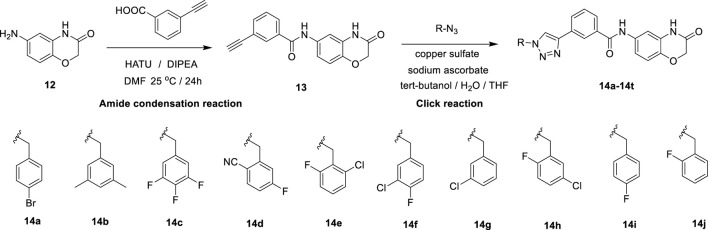
The reaction routes to compounds 14a–14j.

In this route, 6-amino-2H-benzo[b][1,4]oxazin-3(4H)-one (**12)** was condensed with 3-ethynylbenzoic acid under the action of HATU and DIPEA to obtain 3-ethynyl-N-(3-oxo-3,4-dihydro-2H-benzo[b][1,4]oxazin-6-yl)benzamide (**13)**. Compound **13** was reacted with azide compounds of different substituents to obtain 10 novel structure target compounds (**14a-14j**) as shown in Scheme 2. The structures of the target compound were confirmed through ^1^H and ^13^C nuclear magnetic resonance (^1^H NMR and ^13^C NMR) spectroscopy.

## 3 Results and discussion

### 3.1 Inhibitory activity test of 1,2,3-triazole derivatives on IDO1

To evaluate the IDO1 inhibitory activity of the newly synthesized derivatives, HeLa cells with high IDO1 expression were used as the experimental model. Compound 1, designed and synthesized in our previous work (Hou et al., 2022), served as the positive control and showed an IC_50_ value of 0.71 µM, consistent with reported data. Among the tested derivatives, compound 14e exhibited the strongest inhibitory effect with an IC_50_ value of 3.63 µM (Table 1) and was therefore selected for further analgesic studies.

**TABLE 1:** IDO1 inhibitory activities of designed derivatives.

Compounds	IDO1/IC_50_ (μM)	Compounds	IDO1/IC_50_ (μM)
14a	11.23	14f	8.02
14b	>30	14g	10.08
14c	>30	14h	9.98
14d	>30	14i	>30
14e	3.63	14j	>30

### 3.2 Determining the binding modes of the 1,2,3-triazole derivatives on IDO1

To explore the potential binding mode of 1,2,3-triazole derivatives with IDO1, docking simulations were performed. As shown in Figure 3, compound **14e** successfully accessed the active site of the IDO1 enzyme. The 1,2,3-triazole moiety was positioned above the heme, with the nitrogen atom of the triazole group in close proximity to the ferrous ion, facilitating coordination. In addition, the triazole ring formed π-π stacking interactions with PHE163. The 2-fluoro-6-chlorobenzyl group occupied the hydrophobic Pocket A, while the amide bond in the 2H-benzo[b][1,4]oxazin-3(4H)-one scaffold established a hydrogen bond with LYS238 in Pocket B. These interactions highlight the structural basis for the potent IDO1 inhibitory activity of compound **14e** and further support the rationale behind our inhibitor design.

### 3.3 Compound **14e** inhibits the activation of microglia after peripheral inflammation

**FIGURE 4:**
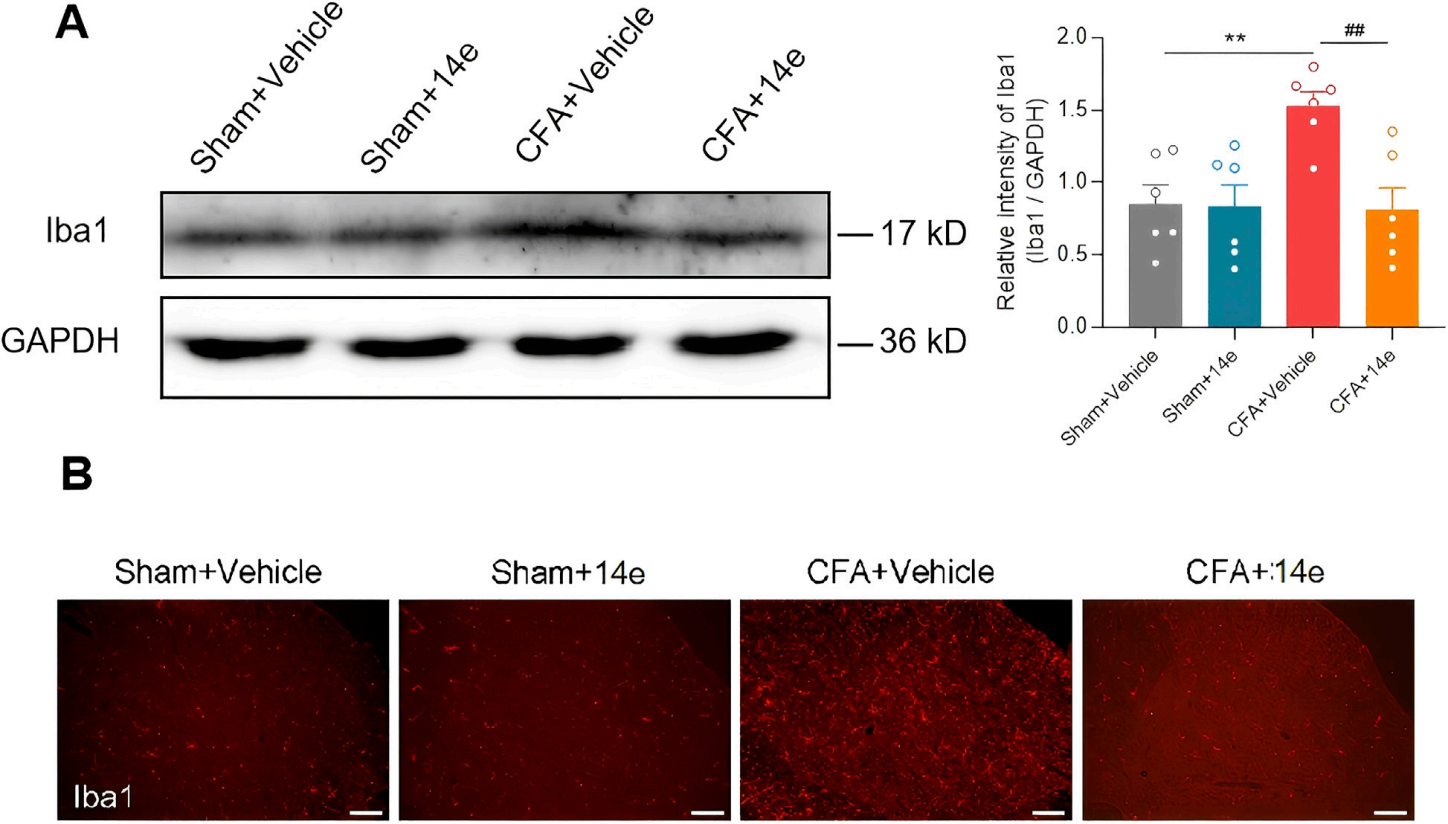
Compound **14e** prevented the upregulation of Iba1 in CFA mice. **(A)** Western blot results indicated that the protein level of Iba1 in sham and CFA mice treated with Vehicle or Compound **14e** had no significant change, while Compound **14e** downregulated the elevated protein expression level of Iba1 induced by CFA in the spinal cord, n = 6 mice/group. **P < 0.01, *versus* the Sham+Vehicle group; ^##^P < 0.01, *versus* the CFA + Vehicle group, unpaired t-test. **(B)** Immunofluorescence staining showed expression of Iba1 was increased in the dorsal horn in mice 3 days after CFA, and Compound **14e** reduced the CFA-induced upregulation of Iba1, Scale bar, 100 μm.

Given the critical role of microglial activation in inflammation-mediated responses, we investigated whether Compound **14e** could modulate microglial activity in the spinal dorsal horn of mice following Complete Freund’s Adjuvant (CFA) injection. First, we assessed the expression of the microglial marker Iba1 in the L4–L6 segments of the spinal cord. Western blot analysis revealed a significant increase in Iba1 protein levels after CFA injection. However, treatment with Compound **14e** significantly reduced the elevated Iba1 expression compared to the SNI+Vehicle group (Figure 4A). Furthermore, the CFA-induced morphological changes and increased accumulation of Iba1-positive microglia in the spinal dorsal horn were notably alleviated by intraperitoneal injection of Compound **14e** (Figure 4B).

### 3.4 Compound **14e** attenuates pain behaviors in the inflammatory pain mouse model

**FIGURE 5:**
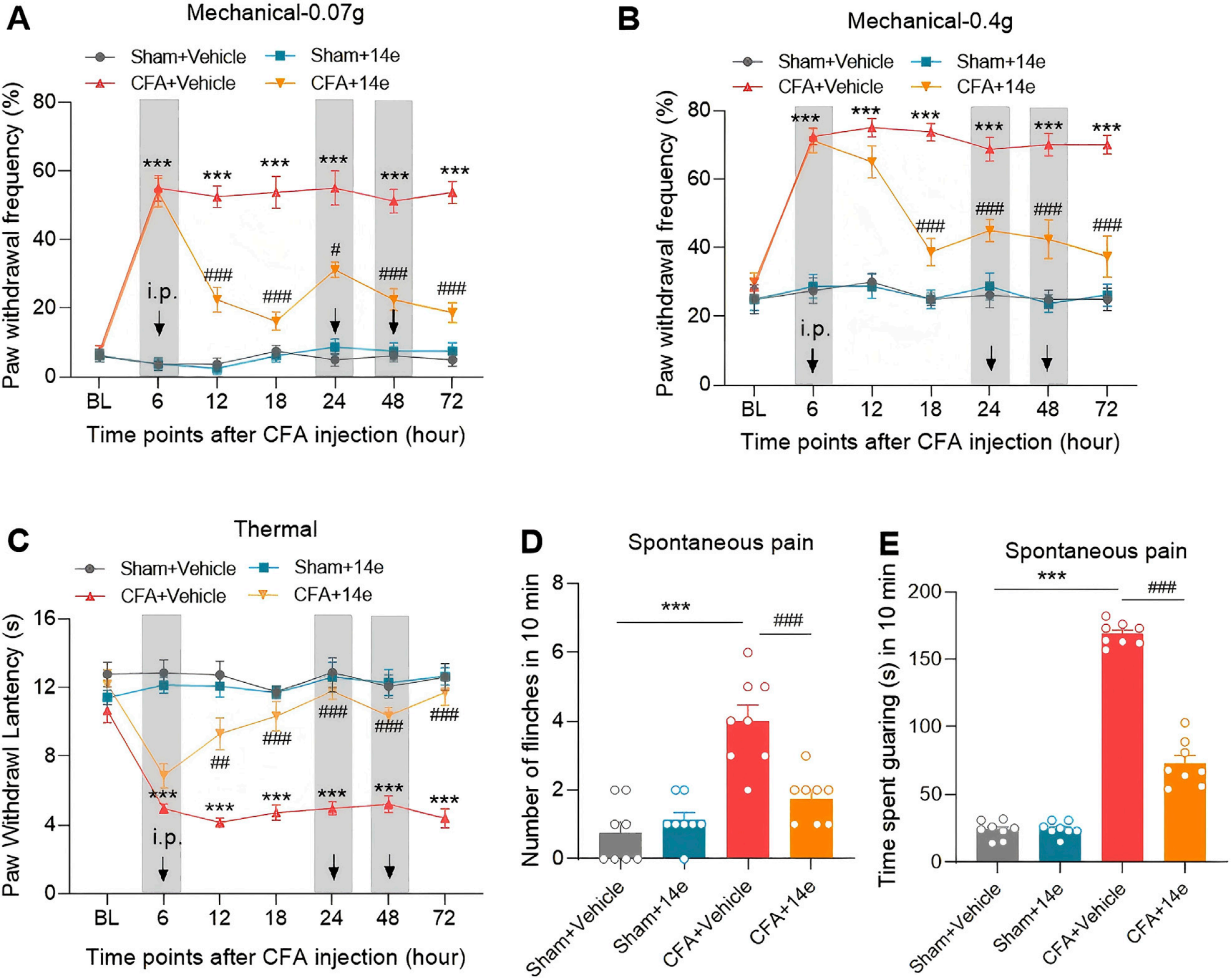
Compound **14e** attenuates mechanical allodynia, thermal hyperalgesia and spontaneous pain of inflammatory pain in mice. **(A,B)** Evaluation of CFA-induced mechanical allodynia as assessed by withdrawal frequency to 0.07 g **(A)** and 0.4 g **(B)** von Frey filaments in mice repetitive treated with 10% DMSO or compound **14e**. **(C)** Assessment of CFA-induced thermal hyperalgesia in the latency in mice treated with 10% DMSO or compound **14e**. **(D,E)** Comparison of spontaneous pain as indicated by flinching behaviors **(D)** or guarding behaviors **(E)** in 10% DMSO or compound **14e**-repetitive treated mice 72 h after CFA injection. n = 8 mice/group. ***P < 0.001, *versus* the Sham+Vehicle group; ^#^P < 0.05, ^##^P < 0.01, ^###^P < 0.001, *versus* the CFA + Vehicle group, two-way ANOVA with Bonferroni’s *post hoc* test **(A–C)** and unpaired t-test **(D,E,G,H)**.

To evaluate the analgesic effects of Compound **14e** *in vivo*, we established an inflammatory pain model using intraplantar injection of CFA. Compound **14e** (30 mg/kg) was administered intraperitoneally at 6 h, with subsequent doses at 48 and 72 h after CFA injection. Behavioral assessments, including von Frey testing for mechanical allodynia and heat stimuli for thermal hyperalgesia, were conducted on the injected hind paw at baseline (BL), as well as at 6, 12, 18, 24, 48 and 72 h post-CFA injection. Additionally, flinching and guarding behaviors, indicators of spontaneous pain, were evaluated 72 h after CFA injection. The results demonstrated that Compound **14e** significantly alleviated mechanical allodynia, as indicated by a reduced paw withdrawal frequency (PWF), and thermal hyperalgesia, as shown by an increased paw withdrawal latency (PWL) on the ipsilateral side from 12 to 72 h post-CFA injection (Figures 5A–C). Furthermore, repeated administration of Compound **14e** significantly reduced spontaneous pain behaviors at 72 h post-CFA injection (Figures 5D,E).

### 3.5 Compound **14e** reduces CFA-induced upregulation of pro-inflammatory cytokines in the inflamed paw, spinal cord and serum

**FIGURE 6:**
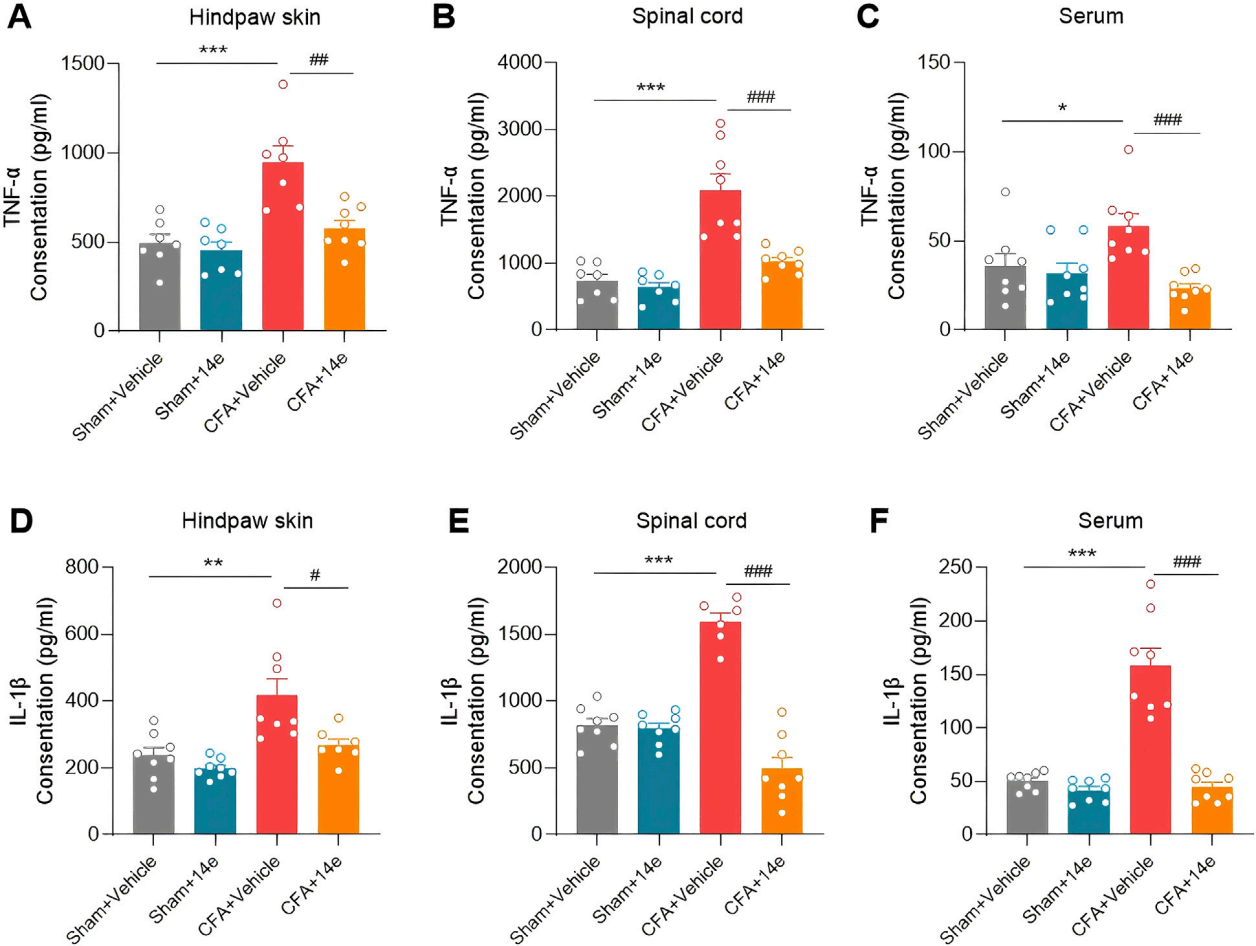
Compound **14e** reduces TNF-α and IL-1β in the inflamed hindpaw skin, spinal cord and peripheral blood 72 h after CFA-induced inflammation. **(A,D)** Expression levels of TNF-α and IL-1β in the inflamed skin tissue. **(B,E)** Expression levels of TNF-α and IL-1β in ipsilateral L4–6 spinal cord. **(C,F)** Expression levels of TNF-α and IL-1β in peripheral serum, n = 6-7 mice/group. *P < 0.05, **P < 0.01, ***P < 0.001, *versus* the Sham+Vehicle group; #P < 0.05, ##P < 0.01, ###P < 0.001, *versus* the CFA + Vehicle group, unpaired t-test.

To determine whether the analgesic effects of Compound **14e** are associated with reduced local and systemic inflammation, we measured the levels of inflammatory factors in the inflamed paw tissue, ipsilateral L4–L6 spinal cord, and peripheral blood. The results indicated a significant increase in the protein levels of TNF-α and IL-1β in the inflamed paw, spinal cord, and serum of mice in the CFA group compared to the vehicle group. Treatment with Compound **14e** significantly decreased the CFA-induced levels of TNF-α (Figures 6A–C) and IL-1β (Figures 6D–F) in these tissues.

### 3.6 Toxicity assay

We evaluated the safety of compound **14e** using an acute toxicity test in KM mice. Male mice (8–9 weeks old) were randomly divided into a solvent control group and a 14e-treated group. The control group received an equivalent volume of solvent (5% DMSO +40% PEG300 + 1% Tween-80 + 54% saline) via gavage, while the treatment group received compound **14e** solution (500 mg/kg). Beginning on day 1 after administration, body weight and general appearance were monitored for 14 days. At the end of the study, blood samples were collected to determine serum levels of Alanine Aminotransferase (ALT) and Aspartate Aminotransferase (AST). In addition, representative organs (heart, liver, spleen, lung, and kidneys) were excised, weighed, and used to calculate organ indices (organ index = organ weight/body weight × 100%).

**FIGURE 7:**
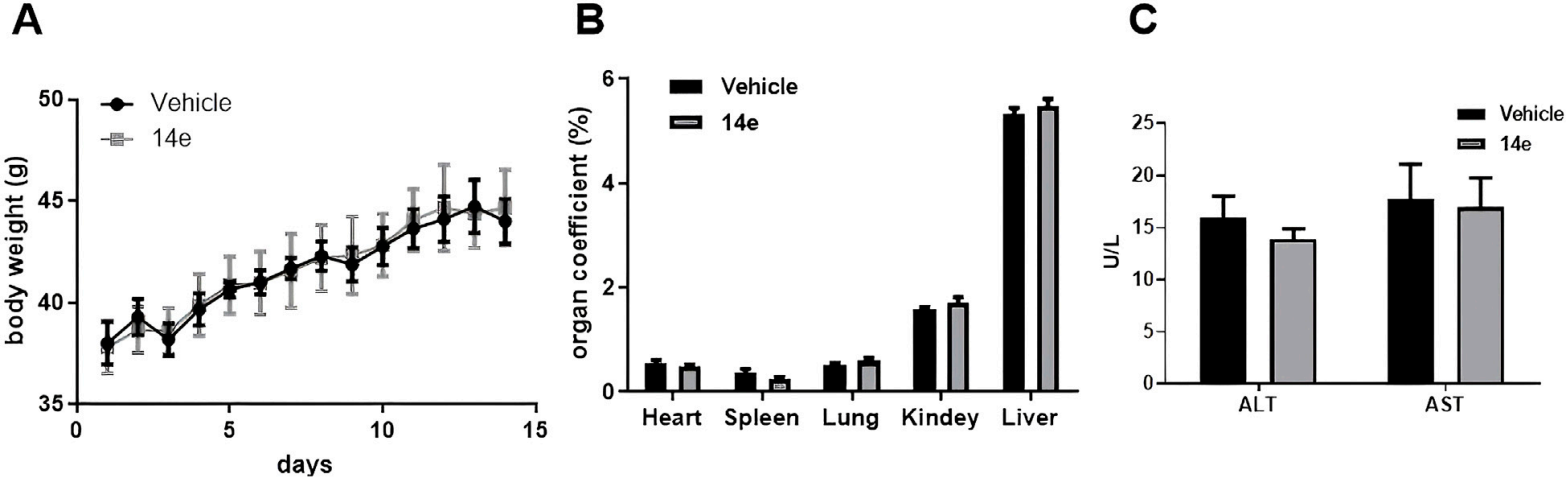
Acute toxicity experiments of compound **14e** were conducted in mice. **(A)** The body wight of mice were recorded for 14 days after treatment. **(B)** The acute toxicity experiments examined the effects of **14e** on mouse organs. **(C)** Effect of acute toxicity experimental studies on blood biochemical indices ALT and AST in mice.

As shown in Figure 7A, no significant changes in body weight were observed between the control and **14e**-treated groups. Similarly, organ indices remained stable across all groups (Figure 7B). Biochemical analysis further demonstrated that treatment with 500 mg/kg of compound **14e** did not significantly affect serum ALT and AST levels (Figure 7C). These results clearly indicate that compound 14e does not cause noticeable toxicity under the tested conditions, supporting its favorable safety profile.

## 4 Conclusion

Building on the structural features of the heme active site in the IDO1 protein crystal, this study designed and synthesized a 2H-benzo[b][1,4]oxazin-3(4H)-one derivative incorporating a 1,2,3-triazole moiety. The experimental results demonstrated that the synthesized compounds generally inhibited IDO1 activity, with compound **14e** exhibiting the most potent effect, achieving an IC_50_ value of 3.63 μM. Molecular docking analysis revealed that the 1,2,3-triazole ring in compound **14e** is positioned directly above the heme’s ferrous ion, forming a metal coordination bond. Additionally, the triazole ring engages in π-π interactions with Phe263, while the amide group in the 2H-benzo[b][1,4]oxazin-3(4H)-one scaffold forms hydrogen bonds with Lys238. In mouse models, compound **14e** significantly reduced the CFA-induced upregulation of Iba1 in the spinal dorsal horn and alleviated inflammatory pain. Furthermore, treatment with compound **14e** markedly decreased the levels of the pro-inflammatory cytokines TNF-α and IL-1β in CFA-treated mice. Notably, compound **14e** exhibited a favorable safety profile, showing no significant toxicity in major organs.

## 5 Experimental

### 5.1 Materials and chemistry

The 2H-1,4-Benzoxazin-3(4H)-one derivatives used in the manuscritp were synthesized previously (Hou et al., 2025). All reagents and solvents obtained from commercially available source were uesd without further treatment. ^1^H NMR and ^13^C NMR spectra were acquired in DMSO-d6 solution with a Bruker 400 spectrometer. High-resolution mass spectra (HRMS) measurements were carried out using a Bruker MicrOTOF-Q Ⅱ mass spectrometer. HPLC uses Agilent’s high performance liquid chromatography. Molecular docking experiments were performed using Schrödinger software. Dulbecco’s modified Eagle medium (DMEM) was obtained from LifeTech (Grand Island, NY, United States). Fetal bovine serum (FBS) was purchased from Gibco (Grand Island, NY, United States). Griess reagent, β-actin antibody and Protease Inhibitor Cocktail were obtained from Sigma-Aldrich (St. Louis, Missouri, United States).

#### 5.1.1 Synthesis of dolutegravir derivatives 14a-14t


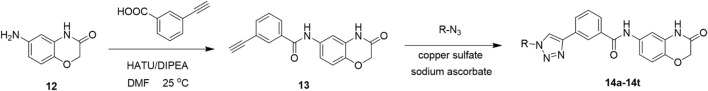


Compound 12 (0.02 mol), 3-aminophenylacetylene (0.03 mol), HATU (0.03 mol), DIPEA (0.06 mol), and 250 mL of DMF were added to a 500 mL reaction flask under nitrogen protection. The mixture was stirred at room temperature for 24 h, with progress monitored by thin-layer chromatography (TLC). After completion of the reaction, indicated by TLC, the solution turned light brown. DMF was removed under reduced pressure, and the residue was extracted three times with 150 mL of dichloromethane. The combined organic layers were washed twice with 150 mL of saturated sodium chloride solution until neutral (pH = 7). The resulting viscous brownish-yellow liquid was concentrated under vacuum. Methanol was then added dropwise under ultrasonic agitation, inducing the formation of a solid precipitate. The mixture was allowed to stand, filtered, and dried to yield Compound 13 (Hou et al., 2025).

In a reaction flask, Compound 13 (3 mmol), substituted azide (3.6 mmol), tert-butanol (70 mL), water (70 mL), and tetrahydrofuran (70 mL) were combined. Anhydrous copper sulfate (6 mmol) and sodium ascorbate (1 mmol) were added, and the reaction mixture was stirred and refluxed at 70 °C for 6 h, with progress monitored by TLC. Upon completion, the reaction mixture was extracted three times with 100 mL of dichloromethane. The combined organic layers were washed twice with 100 mL of saturated sodium chloride solution, followed by two washes with brine (100 mL). The organic layer was dried over sodium sulfate, concentrated under vacuum, and the crude product was obtained. The product was purified by recrystallization from ethyl acetate, yielding the desired compound, which was of sufficient purity for characterization and subsequent anti-tumor studies (Hou et al., 2025).

The spectroscopic characterization of compounds **14a-14t** is provided in Supplementary Material.

### 5.2 Biological study

#### 5.2.1 IDO1 enzymatic inhibitor assay

To perform the HeLa cell-based IDO1 inhibition assay, HeLa cells were seeded at a density of 50,000 cells per well in a 96-well microplate containing 100 μL of DMEM supplemented with 10% fetal bovine serum and 1% penicillin-streptomycin. The cells were incubated overnight at 37 °C in a humidified atmosphere with 5% CO_2_. The following day, 100 μL of growth medium containing diluted inhibitors and 100 ng/mL human IFN-γ was added to each well. The cells were then incubated at 37 °C in a CO_2_ incubator for 18 h. After incubation, 140 μL of culture medium was transferred from each well to a new 96-well plate, followed by the addition of 20 μL of 3.05 N trichloroacetic acid (TCA) to hydrolyze (Hou et al., 2022).N-formylkynurenine. The plate was incubated at 50 °C for 30 min and then centrifuged at 2,500 rpm for 10 min to remove any sediment. Next, 100 μL of the supernatant was transferred to a separate 96-well plate and mixed with 100 μL of 2% (w/v) 4-(dimethylamino)benzaldehyde in acetic acid. After a 10-min incubation at room temperature, the absorbance of the resulting yellow color, indicative of kynurenine, was measured at 480 nm using a microplate reader.

#### 5.2.2 Animals

Male C57BL/6 mice aged 8–10 weeks at the start of experiments were purchased from the laboratory animal center of Zhengzhou University. Animals were housed in a standard 12 h reversed light/dark cycle at 22 °C – 25 °C with free access to food and water. All experimental procedures for animals were carried out according to the relevant guidelines and regulations of the International Association for the Study of Pain and were approved by the Animal Protection and Use Committee of Zhengzhou University (Permit Number: 2023-KY-1173-002).

#### 5.2.3 CFA-induced inflammatory pain model

The mice were anesthetized with isoflurane, and 40 μL of CFA (Sigma-Aldrich) was intraplantarly injected into the mid-plantar surface of their left hind paws. As for the control group, an equivalent volume of normal saline was injected (Jiang et al., 2018).

#### 5.2.4 Drug application

The compound 14e were diluted with 10% DMSO, and were intraperitoneally injected with 30 mg/kg into the mice once a day for 3 days after CFA. The dosage of compound 14e was determined based on our preliminary experiments.

#### 5.2.5 Behavioral tests

Behavioral tests were carried out in a blinded fashion within the time frame of 9:00 a.m. to 4:00 p.m. Each animal was acclimated to a Plexiglas chamber environment for 30 min per day at least 2 consecutive days before testing.

Mechanical hypersensitivity was assessed by measuring paw withdrawal frequency (PWF) as described previously in our reports (Ma et al., 2021; Pan et al., 2021). The PWF in response to mechanical stimulus was determined using two calibrated von Frey filaments (0.07 and 0.4 g, North Coast Medical Inc., Gilroy, United States). Each von Frey filament was applied to stimulate the hindpaw midplantar surface through the mesh floor 10 times at intervals of 5 min. An abrupt paw withdrawal, flinching, or paw licking was regarded as a positive response. The PWF was calculated based on the number of positive responses from the 10 applications ([number of paw withdrawals/10 trials] × 100 = % response frequency).

For the thermal nociceptive response, paw withdrawal latencies (PWLs) to thermal stimulus were measured by a heat flux radiometer (Ugo Basile S.R.L., Italy) (Liu et al., 2023). Briefly, the mouse was placed in an individual Plexiglas chamber on a glass platform. A light beam was emitted and targeted the middle of the plantar surface, with the stimuli cut off if the paw was withdrawn. The duration between the initiation of the light beam and the lifting of the hindfoot was considered as the PWL. The power of the thermal stimulator was adjusted to a cut-off time of 20 s to avoid potential tissue damage. Three latency measurements were taken with at least 5-min intervals for each trial, and the average value was used to represent the thermal pain threshold.

Spontaneous nociceptive behavior was recorded in previous studies (Liu et al., 2023; Yang et al., 2018) Mice were placed in the Plexiglas chamber and allowed to habituate for 30 min. Following acclimatization, the number of flinches and the time spent on guarding behaviors were analyzed for 10 min. Flinching was characterized as the lifting and rapid flexion of the injected hindpaw, and guarding was defined as holding the affected hindpaw elevated in the air (Liu et al., 2021).

#### 5.2.6 Western blot

Animals were deeply anesthetized with 1% pentobarbital sodium (0.1 g/kg, i. p.) and the ipsilateral spinal cord in the lumbar enlargement site was rapidly separated and homogenized in ice-cold lysis buffer containing 50 mM Tris-HCl (pH 7.4), 150 mM NaCl, 1% NP-40, 0.1% SDS, 1 mM phenylmethylsulfonyl fluoride and protease phosphatase inhibitor (Solarbio Company, China). After being kept on ice for 1 h, the lysates were centrifuged at 12,000 rpm for 15 min at 4 °C and the supernatants mixing with loading buffer were denatured at 95 °C for 5 min. The protein concentrations were measured using a bicinchoninic acid (BCA) kit (Pierce Biotechnology Inc.).

According to a standard protocol, an equal amount (40 μg) of protein was separated from each sample on 8%–12% SDS-PAGE and transferred to the polyvinylidene fluoride membrane (Merck Millipore). The blots were blocked in a solution of 5% non-fat milk with TBST (20 mM Tris-HCl, pH 7.6, 150 mM NaCl, 0.05% Tween 20) for 1 h at room temperature and incubated with one of the following primary antibodies at 4 °C overnight: rabbit anti-ionized calcium binding adaptor molecule 1 (Iba1, 1:1,000, DF6442, Affinity Biosciences) or mouse anti-GAPDH (1:50000, 600041-Ig, Proteintech). Subsequently, after washing with TBST 3 times (5 min each time), membranes were incubated with horseradish peroxidase-conjugated goat anti-rabbit secondary antibody (1:10,000, A21020, abbkine) or goat anti-mouse secondary antibody (1:10,000, A21010, abbkine) for 1 h at room temperature. Finally, the blots were detected using ECL reagents and visualized by a ChemiDoc MP System molecular imager (Bio-Rad). The films were analyzed using Quantity One software. Nuclear protein bands were normalized to Histone-H3 and β-tubulin was served as the loading control for total fractions.

#### 5.2.7 Immunohistochemistry

Under anesthesia with 1% pentobarbital sodium (0.1 g/kg, i. p.), mice were perfused intracardially with warm normal saline followed by cold 4% paraformaldehyde (PFA) in 0.1 M phosphate buffer (PB). The whole spinal cord in L4 – L5 segments was rapidly dissected and post-fixed in 4% PFA for 6 h, then cryoprotected by incubation of PB with 20% and 30% sucrose solutions at 4 °C in turn. 25 μm sections were sliced horizontally using a cryostat microtome (CM 1900, Leica). Based on a standard protocol, free-floating sections were washed in PBS, blocked in 10% normal goat serum with 0.3% Triton X-100 in PBS for 1 h at room temperature, and incubated with one of the following primary antibodies: mouse anti-Iba1 (1 : 200, ab283319, Abcam) in 1% bovine serum albumin (BSA) with 0.3% Triton X-100 at 4 °C for 24 h. Sections were then washed in PBS and incubated with one of the following secondary antibodies at room temperature for 2 h: Alexa Fluor 568 conjugated goat anti-mouse IgG (1 : 200, A-11004, Thermo Fisher Scientific), rinsed in PBS, then dried and covered with anti-fade mounting medium. Images were taken with a fluorescence microscope (BX53, Olympus).

#### 5.2.8 Enzyme-linked immunosorbent assay analysis (ELISA)

Under anesthesia with 1% pentobarbital sodium, serum was obtained from the mice orbits, after coagulation at room temperature for 2 h, the sample was then centrifuged at 1,000 × g for 20 min at 4 °C, with the supernatant collected. Right hippocampus, left L4–6 spinal segments and the central plantar surface of left (injected) hindpaws tissues were homogenized with RIPA lysis buffer at 5,000×g for 5 min at 4 °C and the supernatants were collected. The protein concentrations were determined using a bicinchoninic acid (BCA) kit (Pierce Biotechnology Inc.). The following inflammatory factors were measured using Mouse TNF-α ELISA kit (EM0183) and IL-1β ELISA kit (EM0109) obtained from Wuhan Fine Biotechnology Company, according to the product protocols. The O.D. absorbance at 450 nm was quantified in a ultramicroplate spectrophotometer (BioTek Epoch).

#### 5.2.9 Hematoxylin-eosin (HE) staining

In this study, we performed HE staining to analyze the liver, kidney, brain, heart, and lung tissues. Tissue samples were fixed in 4% paraformaldehyde (PFA) overnight. After dehydration with increasing concentrations of ethanol, the tissues were embedded in paraffin wax and sectioned at a thickness of 5–7 μm. The sections were mounted on glass slides and deparaffinized using xylene substitutes followed by rehydration through descending ethanol concentrations. Subsequently, the sections were stained with hematoxylin to visualize nuclei, differentiated with acid alcohol, and counterstained with eosin to highlight cytoplasmic components. After dehydration and clearing, the mounted slides were observed under a DFC365FX microscope (Leica).

#### 5.2.10 System preparation

The crystallographic structure of indoleamine 2,3-dioxygenase 1 (IDO1) (PDB ID: 4PK6) with a resolution of 3.45 Å was retrieved from the Protein Data Bank (https://www.rcsb.org). The initial structure was refined using the “Protein Preparation Wizard” tool in Maestro to generate a suitable model for subsequent computational analysis (Sastry et al., 2013). Ligand structure (14e) was optimized using the LigPrep module in Maestro. Considering the coordinate of molecules could form via a N or O atom with the heme, the spherical regions that should be occupied by the Fe atom of heme during docking was chosen as constraints in the Receptor Grid Generation of maestro (Lewis-Ballester et al., 2017). The docking site was defined by a grid box generated automatically using the Receptor Grid Generation tool, based on the ligand-binding site of IDO1. The resulting grid file was utilized for molecular docking to explore potential binding modes of the prepared ligands with IDO1.

#### 5.2.11 Statistical analyses

Data were presented as means ± SEM and performed using Graph Prim 7.0. A two-tailed Student’s t-test or one-way analysis of variance followed by a Student-Newman-Keuls (SNK) test were used to assess significant differences. P < 0.05 was considered statistically significant.

## Data Availability

The datasets presented in this study can be found in online repositories. The names of the repository/repositories and accession number(s) can be found in the article/Supplementary Material.
