# Supplementary material for: Rational design of indoleamine 2,3-dioxygenase 1 (IDO1) inhibitors featuring 1,2,3-triazole derivatives with enhanced anti-inflammatory and analgesic efficacy
Source: Front Pharmacol. 2025 Sep 19;16:1574007. doi: 10.3389/fphar.2025.1574007 (PMC12491243; doi:10.3389/fphar.2025.1574007)

**Supplementary materials**

The spectroscopic characterization of compounds **14a-14t** is provided as follow.

Compound 14a: ^1^H NMR(400MHz, DMSO-d_6_): 10.78 (s, 1H, NH), 10.29 (s, 1H, NH), 8.72 (s, 1H, CH), 8.38 (s, 1H, Ar-H), 8.04 (d, J=8.0Hz, 1H, Ar-H), 7.88 (d, J=8.0Hz, 1H, Ar-H), 7.62-7.53 (m, 4H, Ar-H), 7.34 (d, J=8.0Hz, 2H, Ar-H), 7.26 (d, J=12.0Hz, 1H, Ar-H), 6.93 (d, J=12.0Hz, 1H, Ar-H), 5.67 (s, 2H, CH2), 4.55 (s, 2H, CH2). ^13^C NMR(100MHz, DMSO-d_6_): 165.55, 146.65, 139.94, 136.18, 135.79, 134.30, 132.24, 131.25, 130.71, 129.49, 128.50, 127.60, 127.56, 124.88, 122.57, 121.98, 116.40, 115.66, 109.00, 67.32, 52.86.

Compound 14b: ^1^H NMR(400MHz, DMSO-d_6_): 10.78 (s, 1H, NH), 10.29 (s, 1H, NH), 8.69 (s, 1H, CH), 8.39 (s, 1H, Ar-H), 8.05 (d, J=8.0Hz, 1H, Ar-H), 7.88 (d, J=8.0Hz, 1H, Ar-H), 7.59-7.54 (m, 2H, Ar-H), 7.26 (d, J=12.0Hz, 1H, Ar-H), 6.98-6.92 (m, 4H, Ar-H), 5.58 (s, 2H, CH2), 4.55 (s, 2H, CH2), 2.26 (s, 6H, 2CH3). ^13^C NMR(100MHz, DMSO-d_6_): 165.55, 146.56, 139.94, 138.43, 136.16, 134.30, 131.33, 130.08, 130.00, 129.47, 128.48, 127.60, 127.50, 126.57, 126.18, 124.84, 122.42, 116.40, 115.66, 109.01, 67.32, 53.61, 21.30.

Compound 14c: ^1^H NMR(400MHz, DMSO-d_6_): 10.78 (s, 1H, NH), 10.30 (s, 1H, NH), 8.74 (s, 1H, CH), 8.38 (s, 1H, Ar-H), 8.04 (d, J=8.0Hz, 1H, Ar-H), 7.89 (d, J=12.0Hz, 1H, Ar-H), 7.62-7.58 (m, 1H, Ar-H), 7.54 (s, 1H, Ar-H), 7.43-7.39 (m, 2H, Ar-H), 7.27-7.24 (m, 1H, Ar-H), 6.94 (d, J=8.0Hz, 1H, Ar-H), 5.58 (s, 2H, CH2), 4.55 (s, 2H, CH2). ^13^C NMR(100MHz, DMSO-d_6_): 165.55, 146.67, 139.94, 136.20, 134.29, 131.19, 129.48, 128.53, 127.58 (d, *J*=3.0Hz), 124.94, 122.68, 113.82 (d, *J*=6.0Hz), 113.66 (d, *J*=6.0Hz), 108.99, 67.31, 60.21, 52.19, 21.22, 14.55.

Compound 14d: ^1^H NMR(400MHz, DMSO-d_6_): 10.78 (s, 1H, NH), 10.31 (s, 1H, NH), 8.77 (s, 1H, CH), 8.39 (s, 1H, Ar-H), 8.07-8.05 (m, 2H, Ar-H), 7.90 (d, J=12.0Hz, 1H, Ar-H), 7.63-7.59 (m, 1H, Ar-H), 7.5-7.41 (m, 3H, Ar-H), 7.27-7.24 (m, 1H, Ar-H), 6.94 (d, J=8.0Hz, 1H, Ar-H), 5.91 (s, 2H, CH2), 4.55 (s, 2H, CH2). ^13^C NMR(100MHz, DMSO-d_6_): 165.56, 146.59, 142.49, 142.40, 139.94, 137.01, 136.91, 136.22, 134.30, 131.10, 129.50, 128.58, 127.60, 124.97, 123.05, 118.02, 117.78, 117.49, 117.27, 116.74, 116.40, 115.66, 109.00, 108.50 (d, *J*=3.0Hz), 67.32, 51.50

Compound 14e: ^1^H NMR(400MHz, DMSO-d_6_): 10.78 (s, 1H, NH), 10.28 (s, 1H, NH), 8.69 (s, 1H, CH), 8.38 (s, 1H, Ar-H), 8.06 (d, J= 8.0Hz, 1H, Ar-H), 7.87 (d, J=8.0Hz, 1H, Ar-H), 7.60-7.50 (m, 3H, Ar-H), 7.46-7.35 (m, 2H, Ar-H), 7.27-7.24 (m, 1H, Ar-H), 6.94 (d, J=12.0Hz, 1H, Ar-H), 5.79 (s, 2H, CH2), 4.55 (s, 2H, CH2). ^13^C NMR(100MHz, DMSO-d_6_): 165.55, 146.24, 139.93, 136.20, 135.48, 134.30, 132.43, 132.33, 131.16, 129.43, 128.58, 127.57 (d, *J*=3.0Hz), 126.40 (d, *J*=3.0Hz), 124.93, 122.61, 116.40, 115.63 (d, *J*=5.0Hz), 115.39, 109.00, 67.32, 45.22

Compound 14f: ^1^H NMR(400MHz, DMSO-d_6_): 10.78 (s, 1H, NH), 10.30 (s, 1H, NH), 8.75 (s, 1H, CH), 8.38 (s, 1H, Ar-H), 8.05 (d, J= 8.0Hz, 1H, Ar-H), 7.89 (d, J=8.0Hz, 1H, Ar-H), 7.69-7.67 (m, 1H, Ar-H), 7.60 (t, J=8.0Hz, 1H, Ar-H), 7.54 (s, 1H, Ar-H), 7.48-7.40 (m, 2H, Ar-H), 7.25 (dd, J_1_=4.0Hz, J_2_=4.0Hz, 1H, Ar-H), 6.94 (d, J=8.0Hz, 1H, Ar-H), 5.69 (s, 2H, CH2), 4.55 (s, 2H, CH2). ^13^C NMR(100MHz, DMSO-d_6_): 165.55, 146.67, 139.94, 136.19, 134.30, 130.98, 129.61, 129.53, 129.48, 128.52, 127.57, 124.90, 122.57, 120.20 (d, *J*=18.0Hz), 118.37, 117.94, 117.73, 116.40, 115.65, 109.00, 67.32, 52.24

Compound 14g:^1^H NMR(400MHz, DMSO-d_6_): 10.78 (s, 1H, NH), 10.30 (s, 1H, NH), 8.76 (s, 1H, CH), 8.39 (s, 1H, Ar-H), 8.05 (d, J= 12.0Hz, 1H, Ar-H), 7.89 (d, J=8.0Hz, 1H, Ar-H), 7.60 (t, J_1_=8.0Hz, J_2_=8.0Hz, 1H, Ar-H), 7.54 (s, 1H, Ar-H), 7.48-7.43 (m, 3H, Ar-H), 7.35-7.32 (m, 1H, Ar-H), 7.26 (dd, J_1_=4.0Hz, J_2_=4.0Hz, 1H, Ar-H), 6.94 (d, J=8.0Hz, 1H, Ar-H), 5.71 (s, 2H, CH2), 4.55 (s, 2H, CH2). ^13^C NMR(100MHz, DMSO-d_6_): 165.55, 146.67, 139.94, 138.75, 136.19, 134.30, 133.84, 131.25, 131.23, 129.49, 128.70, 128.52, 128.38, 127.60, 127.58, 127.19, 124.90, 122.66, 116.40, 115.65, 109.00, 67.32, 52.83.

Compound 14h: ^1^H NMR(400MHz, DMSO-d_6_): 10.78 (s, 1H, NH), 10.30 (s, 1H, NH), 8.74 (s, 1H, CH), 8.39 (s, 1H, Ar-H), 8.07 (s, 1H, Ar-H), 7.88 (s, 1H, Ar-H), 7.61-7.25 (m, 6H), Ar-H, 6.94 (d, J=8.0Hz, 1H, Ar-H), 5.74 (s, 2H, CH2), 4.55 (s, 2H, CH2). ^13^C NMR(100MHz, DMSO-d_6_): 165.56, 160.72, 158.26, 146.55, 139.94, 138.59, 136.20, 134.30, 131.15, 131.06, 129.49, 129.01, 128.55, 127.60, 125.20 (d, *J*=17.0Hz), 124.92, 122.69, 118.22 (d, *J*=24.0Hz), 116.40, 115.67, 109.02, 67.32, 47.32

Compound 14i: ^1^H NMR(400MHz, DMSO-d_6_): 10.81 (s, 1H, NH), 10.31 (s, 1H, NH), 8.73 (s, 1H, CH), 8.38 (s, 1H, Ar-H), 8.06-8.04 (m, 1H, Ar-H), 7.88 (d, J=8.0Hz, 1H, Ar-H), 7.62-7.44 (m, 4H, Ar-H), 7.27-7.22 (m, 3H, Ar-H), 6.94 (d, J=8.0Hz, 1H, Ar-H), 5.68 (s, 2H, CH2), 4.56 (s, 2H, CH2). ^13^C NMR(100MHz, DMSO-d_6_): 165.58, 163.64, 161.21, 146.64, 139.83, 136.15, 135.87, 134.29, 132.62, 131.26, 129.50, 128.49, 127.58, 124.85, 122.44, 116.34 (d, *J*=17.0Hz), 116.04, 115.65, 108.98, 67.30, 52.80

Compound 14j: ^1^H NMR(400MHz, DMSO-d_6_): 10.78 (s, 1H, NH), 10.29 (s, 1H, NH), 8.71 (s, 1H, CH), 8.39 (s, 1H, Ar-H), 8.04 (d, J=8.0Hz, 1H, Ar-H), 7.88 (d, J=8.0Hz, 1H, Ar-H), 7.61-7.26 (m, 7H, Ar-H), 6.94 (d, J=8.0Hz, 1H, Ar-H), 5.74 (s, 2H, CH2), 4.55 (s, 1H, CH2). ^13^C NMR(100MHz, DMSO-d_6_): 165.56, 146.52, 139.94, 136.18, 134.30, 131.35, 131.27, 131.23, 129.48, 128.54, 127.60, 127.57, 125.40 (d, *J*=3.0Hz), 124.89, 123.24, 123.09, 122.59, 116.33 (d, *J*=14.0Hz), 116.05, 115.67, 109.02, 67.32, 47.69

# Figure 1. ^1^H NMR and ^13^C NMR spectrums of compound 13


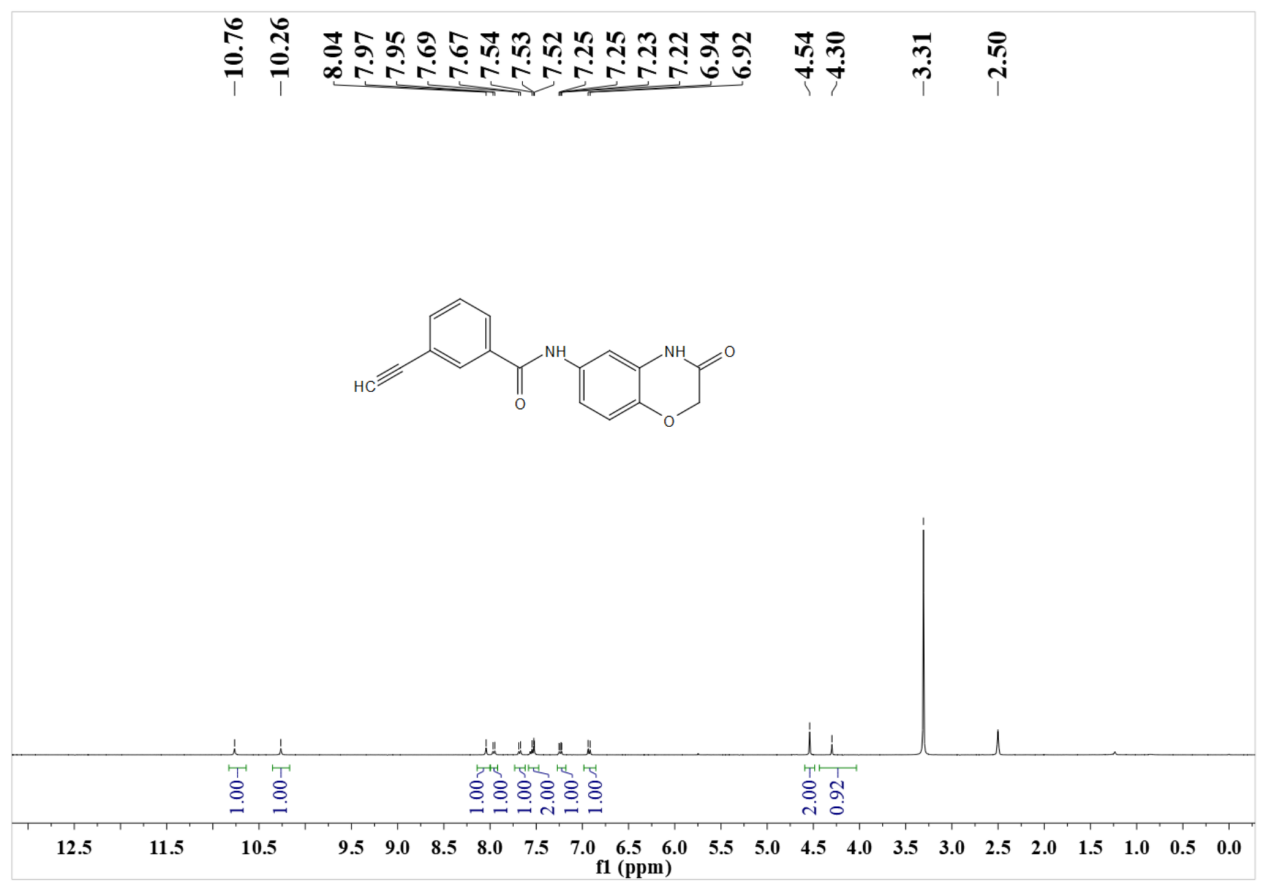


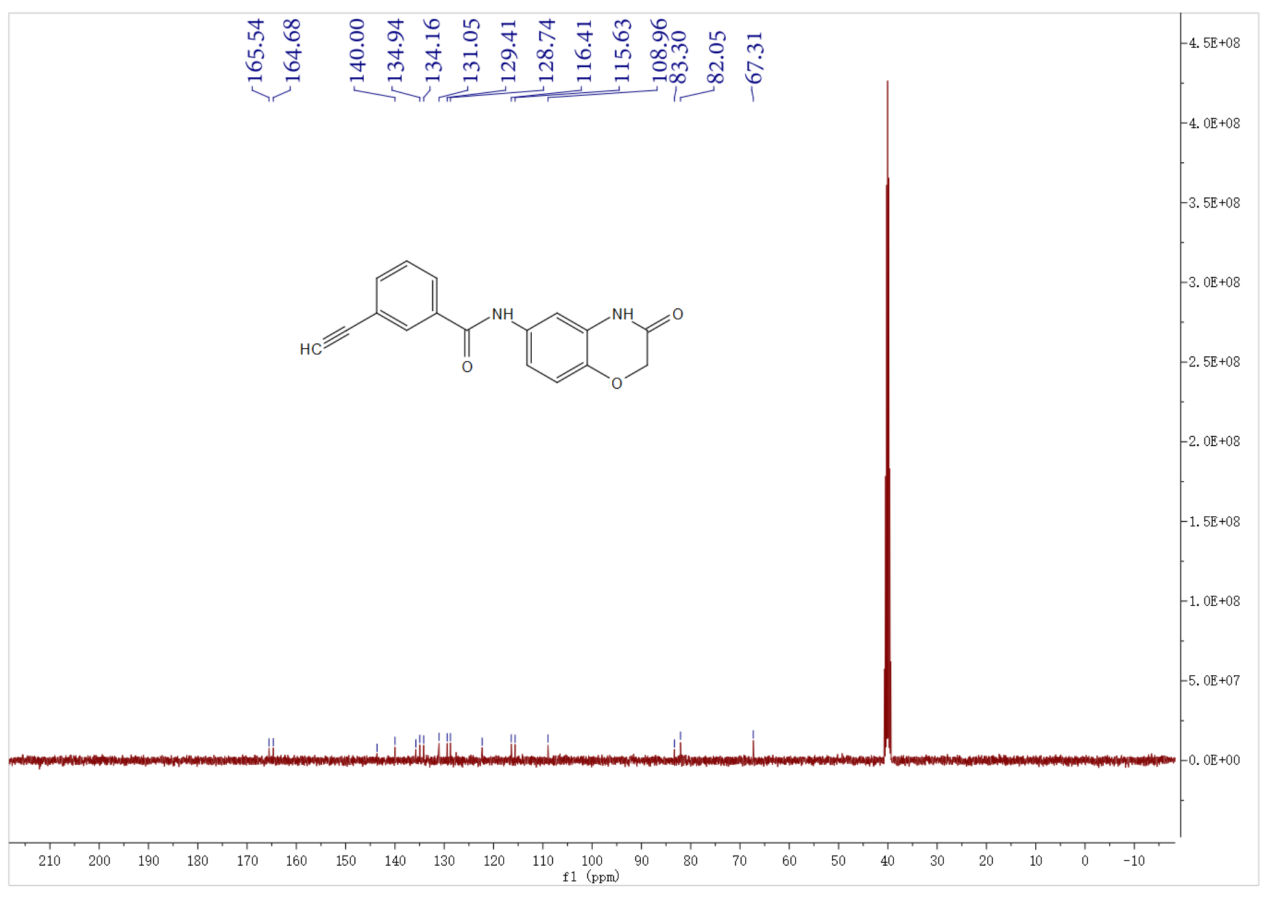


# Figure 2. ^1^H NMR and ^13^C NMR spectrums of compound 14a


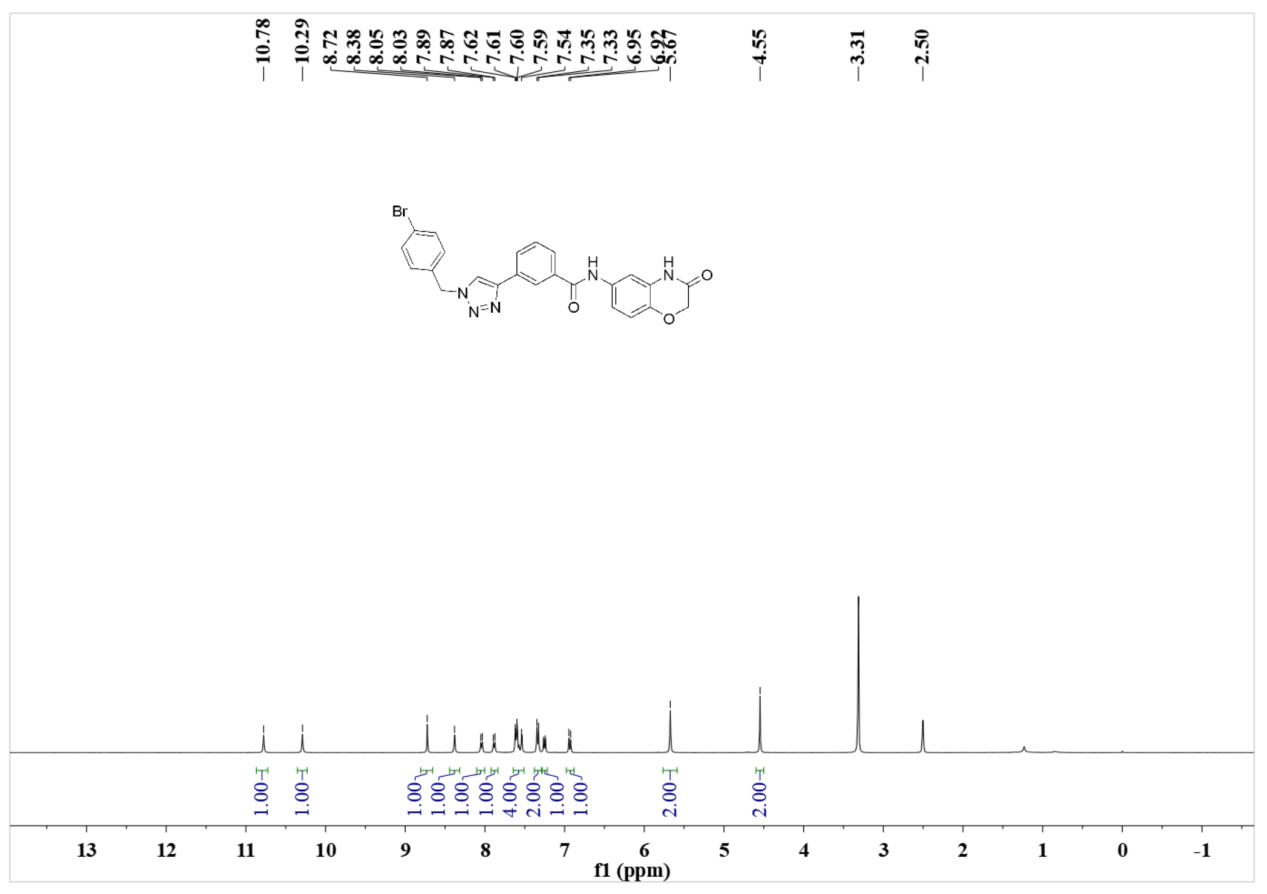


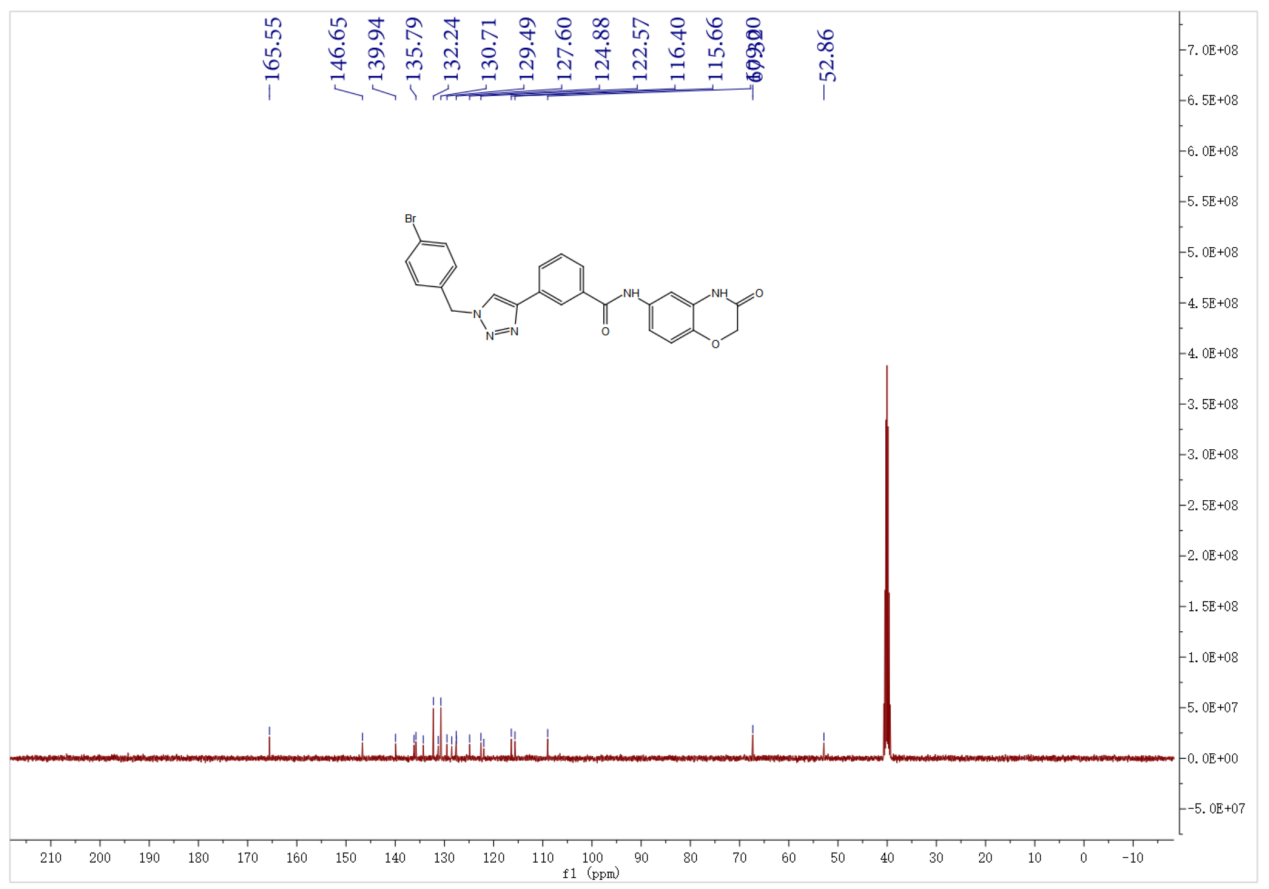


# Figure 3. ^1^H NMR and ^13^C NMR spectrums of compound 14b


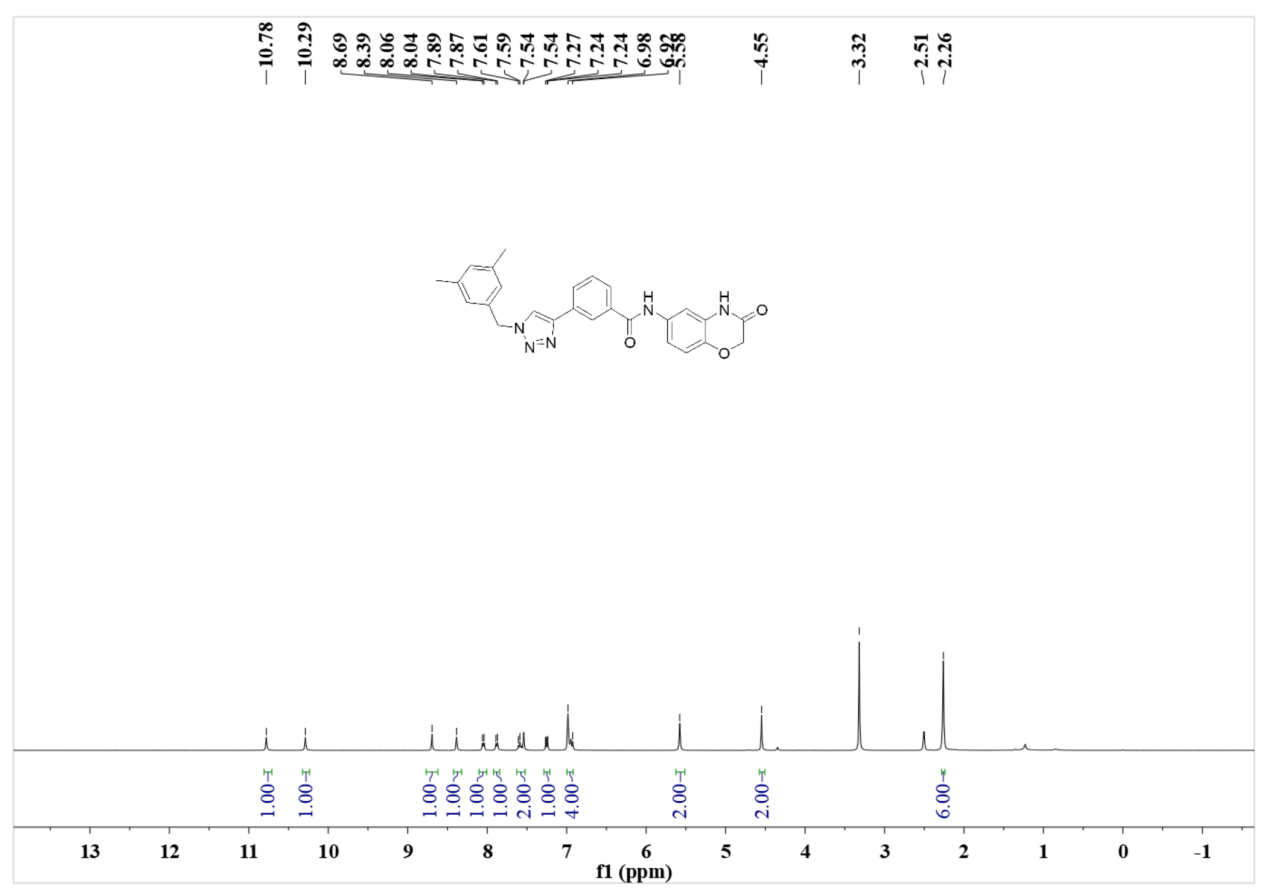


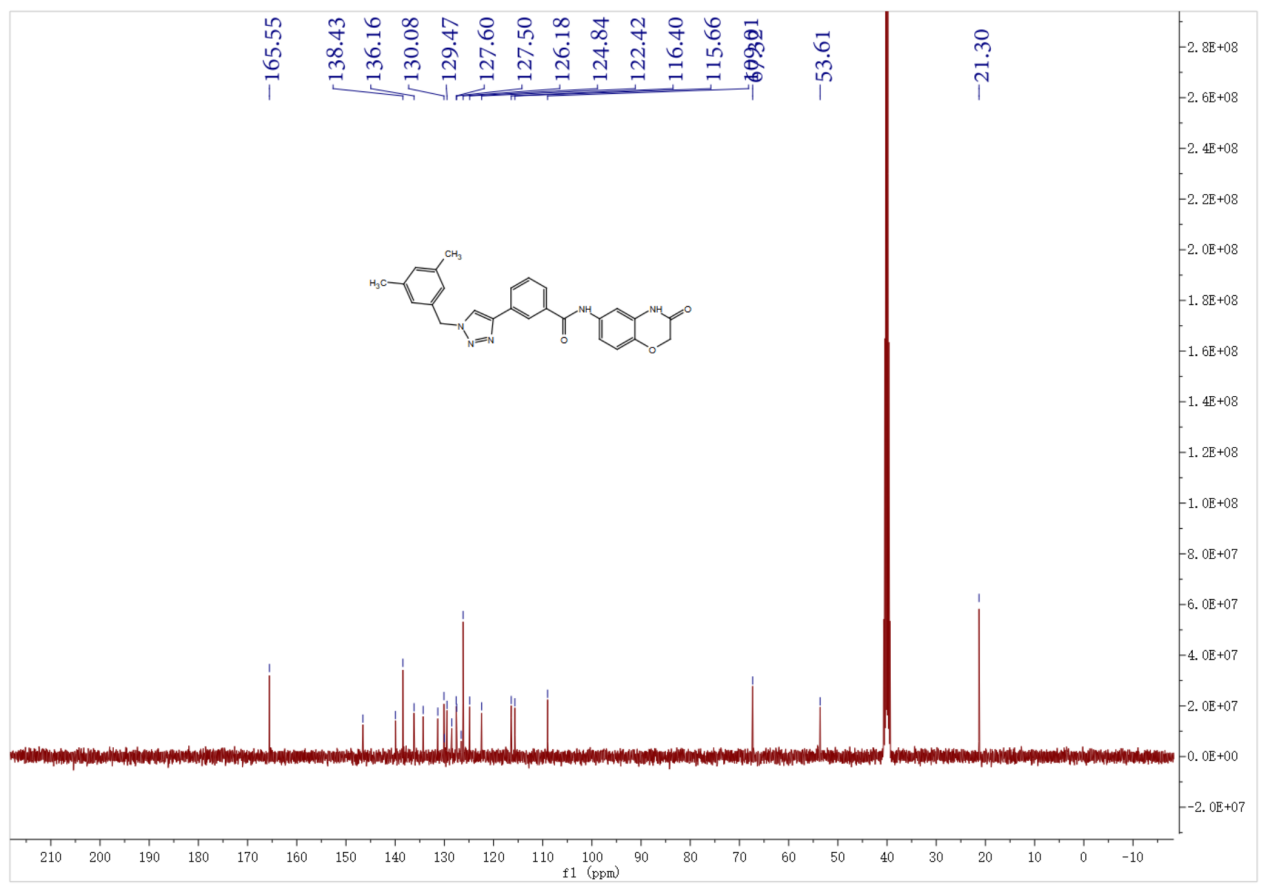


# Figure 4. ^1^H NMR and ^13^C NMR spectrums of compound 14c


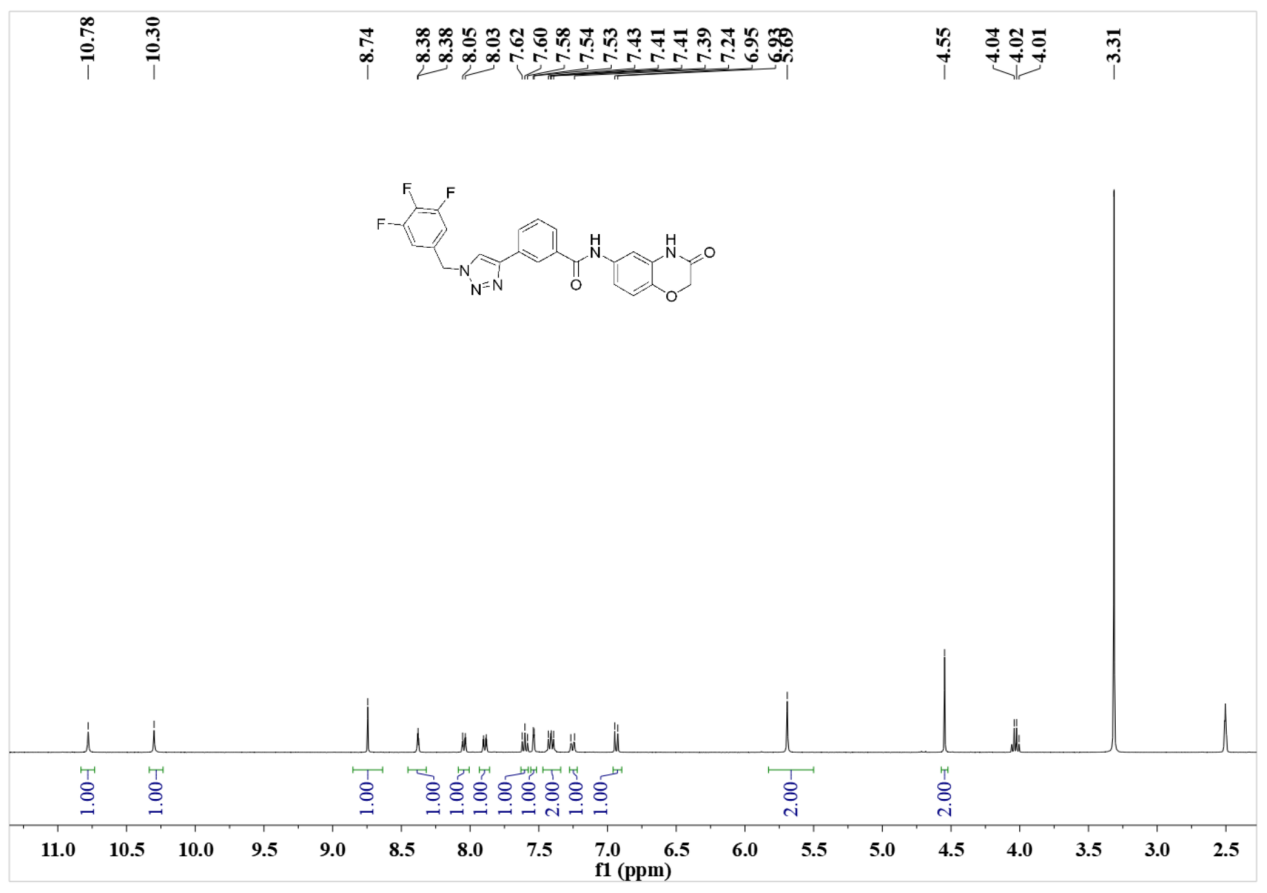


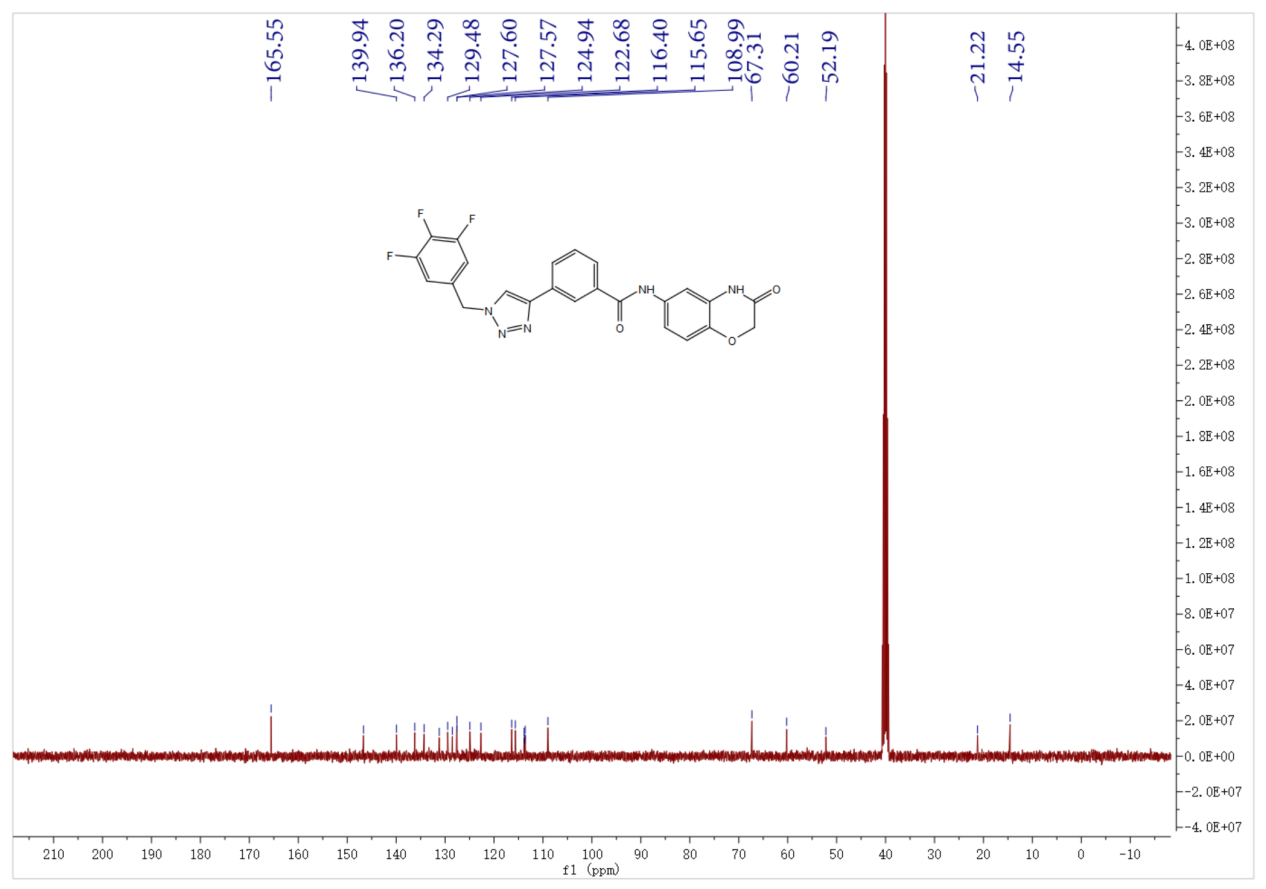


# Figure 5. ^1^H NMR and ^13^C NMR spectrums of compound 14d


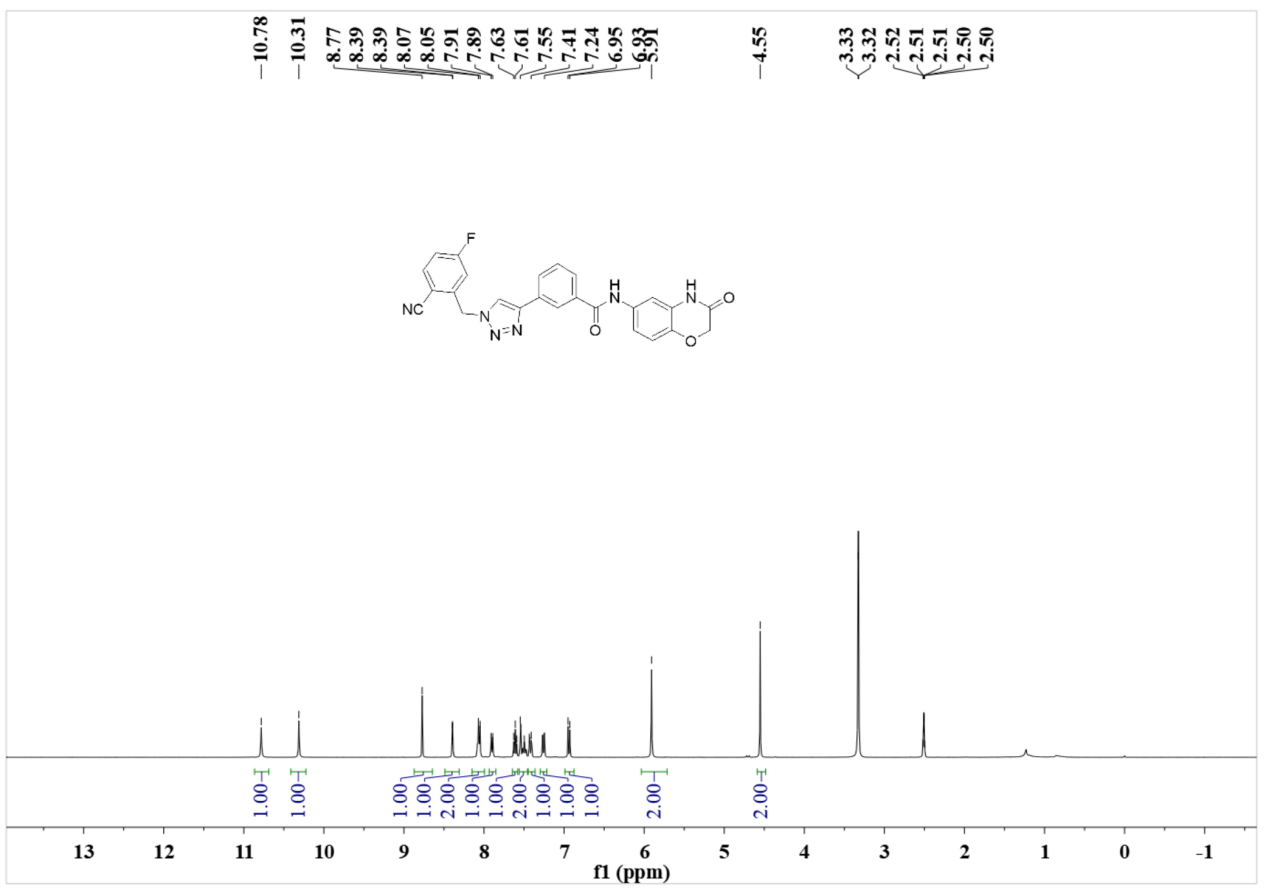


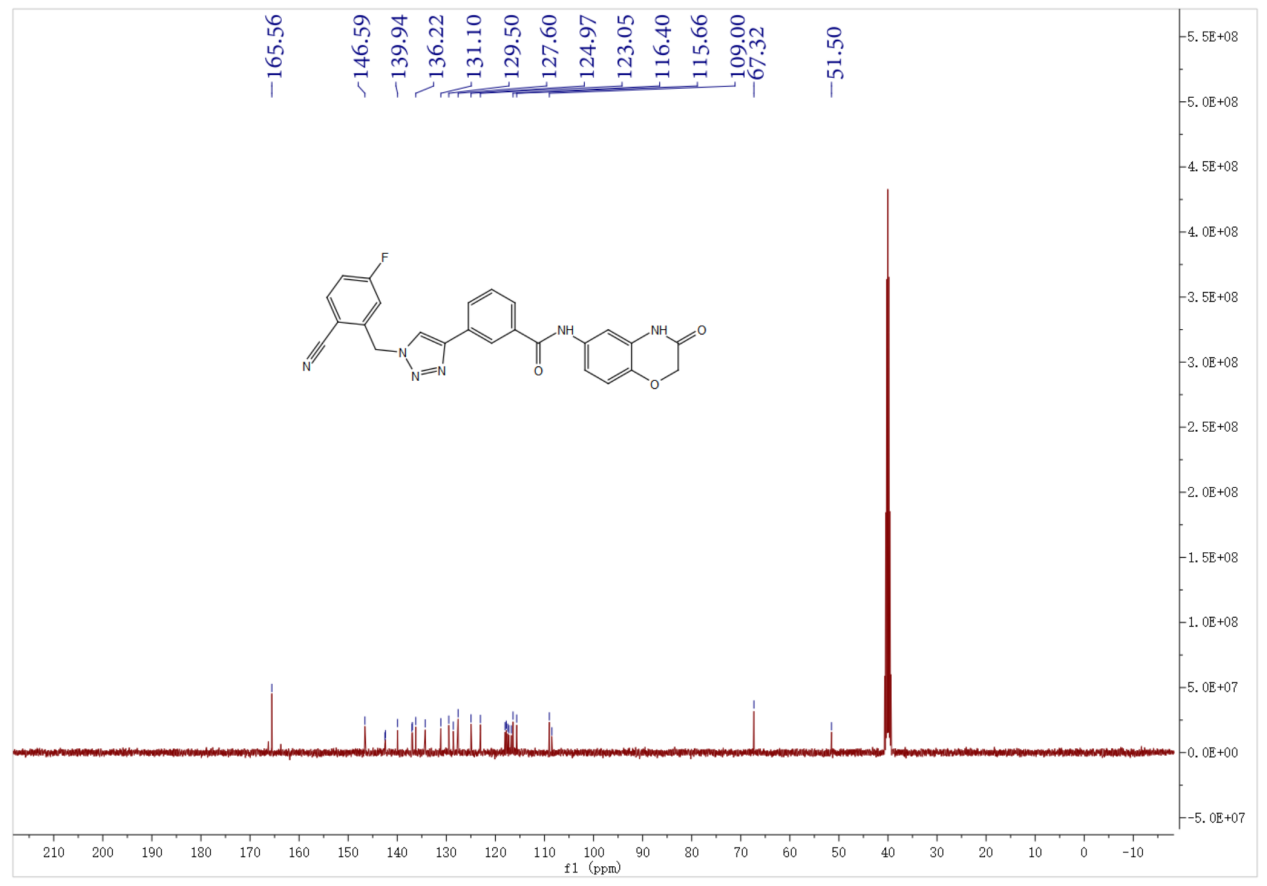


# Figure 6. ^1^H NMR and ^13^C NMR spectrums of compound 14e


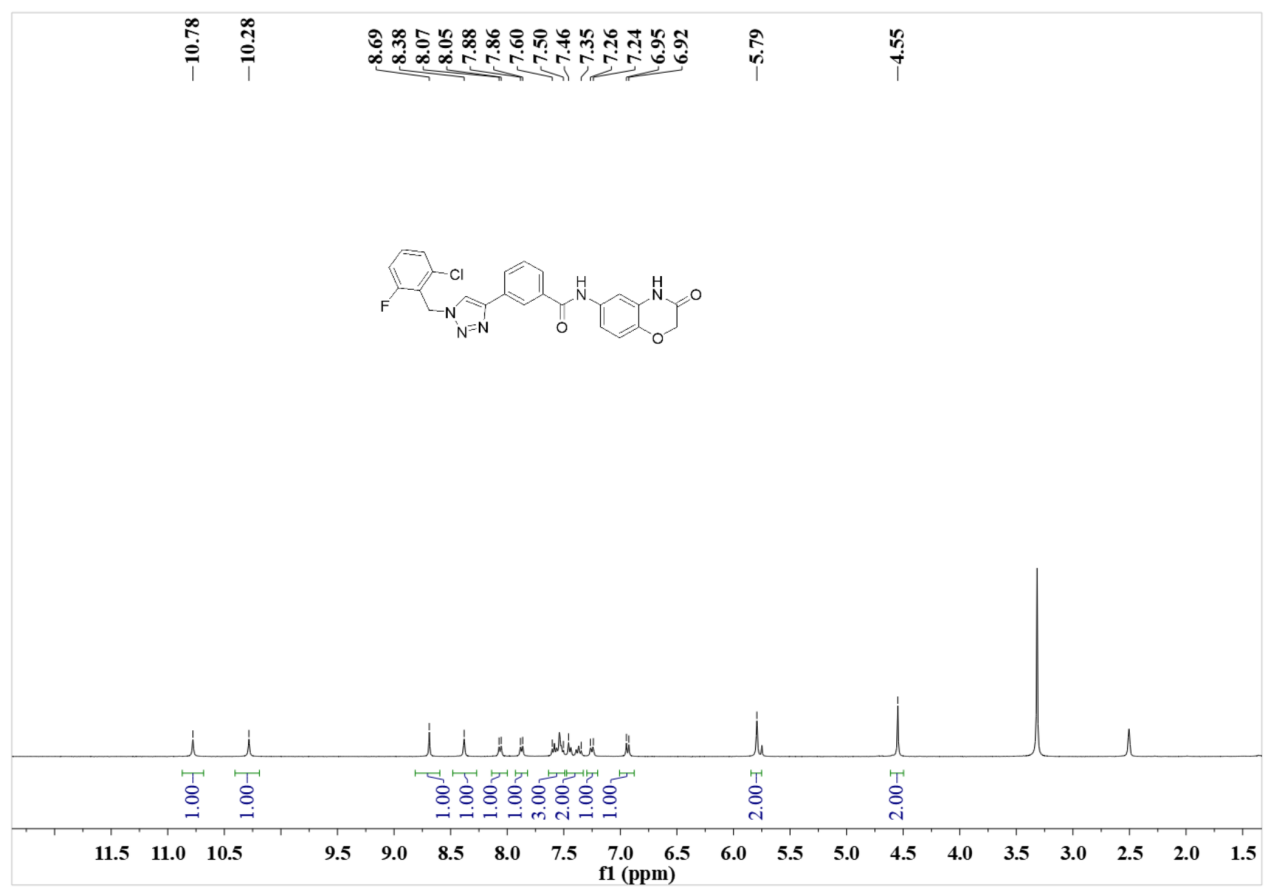


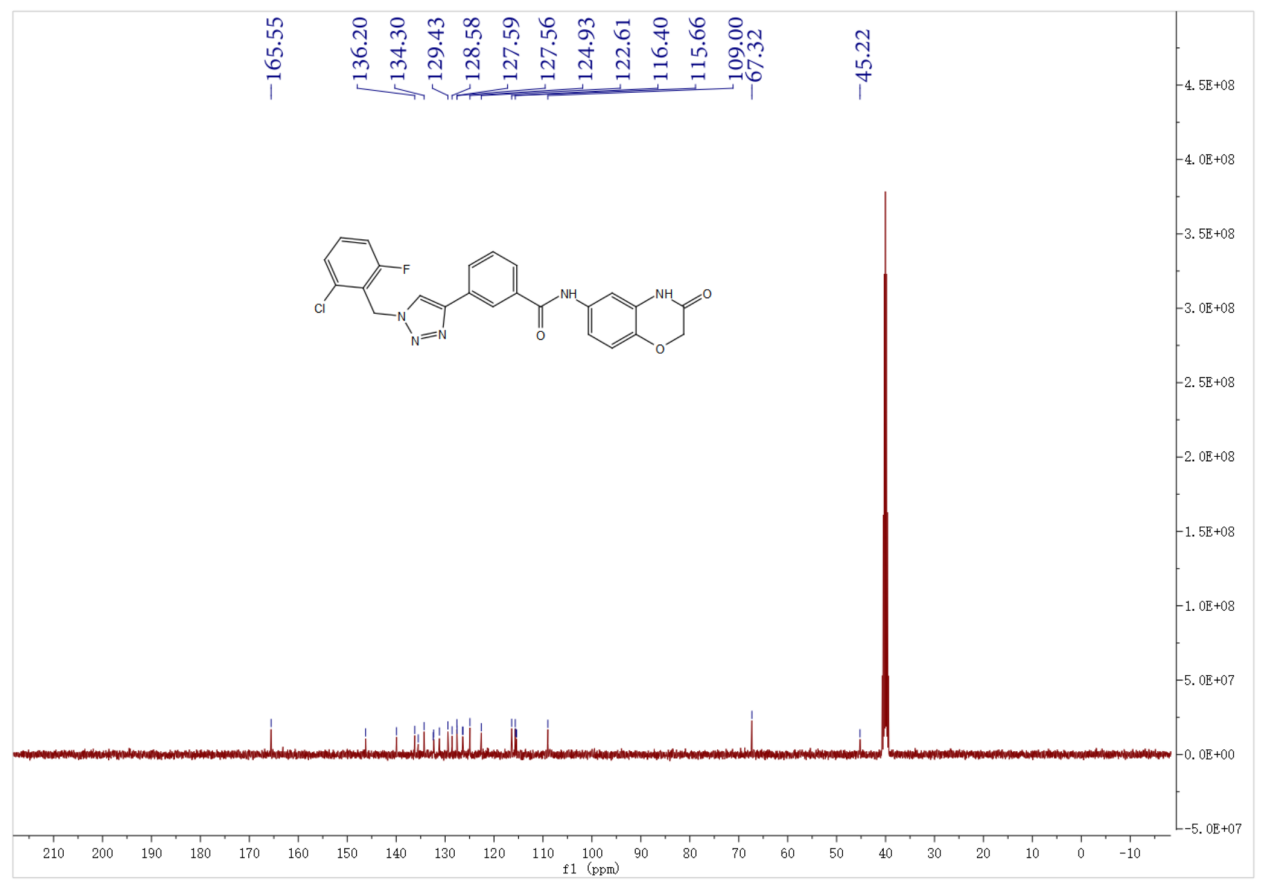


# Figure 7. ^1^H NMR and ^13^C NMR spectrums of compound 14f


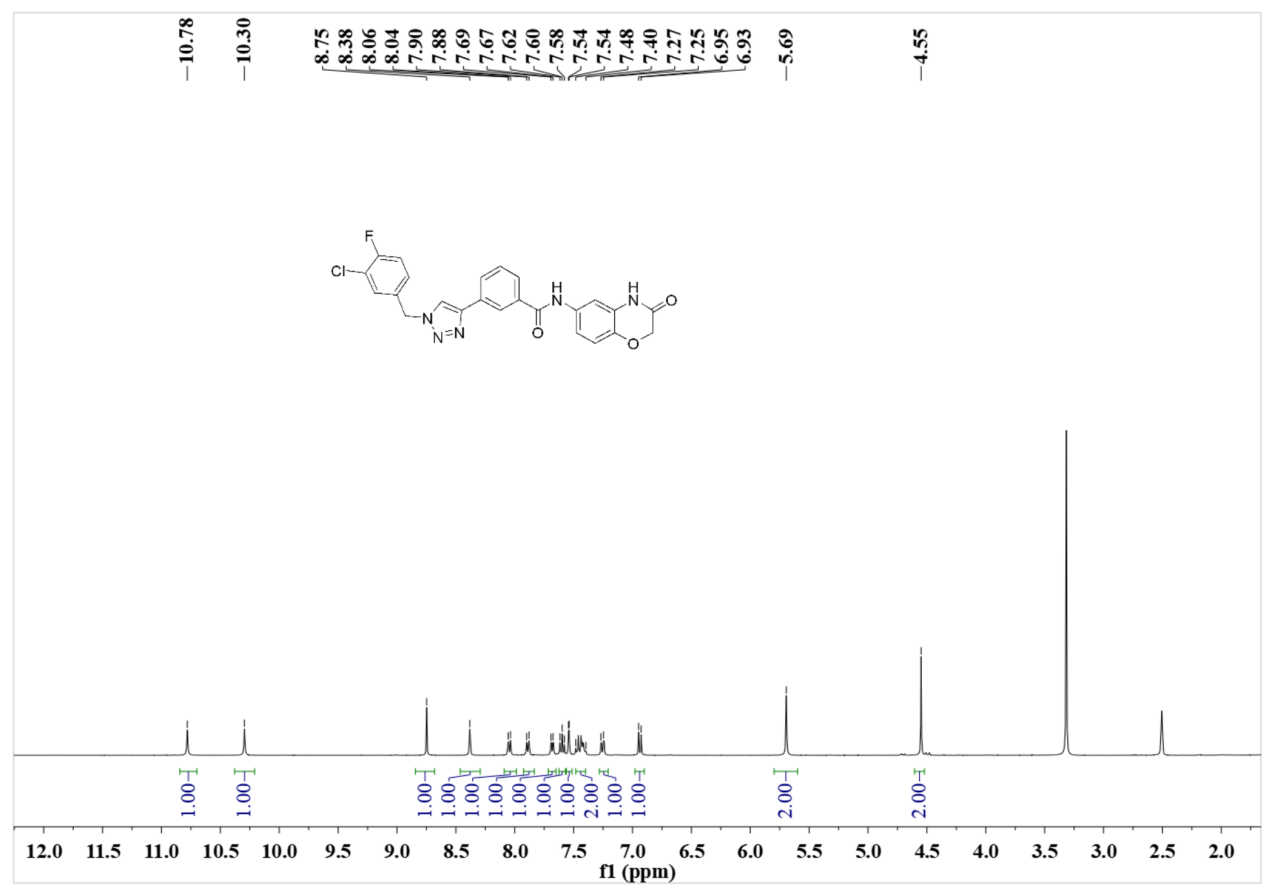


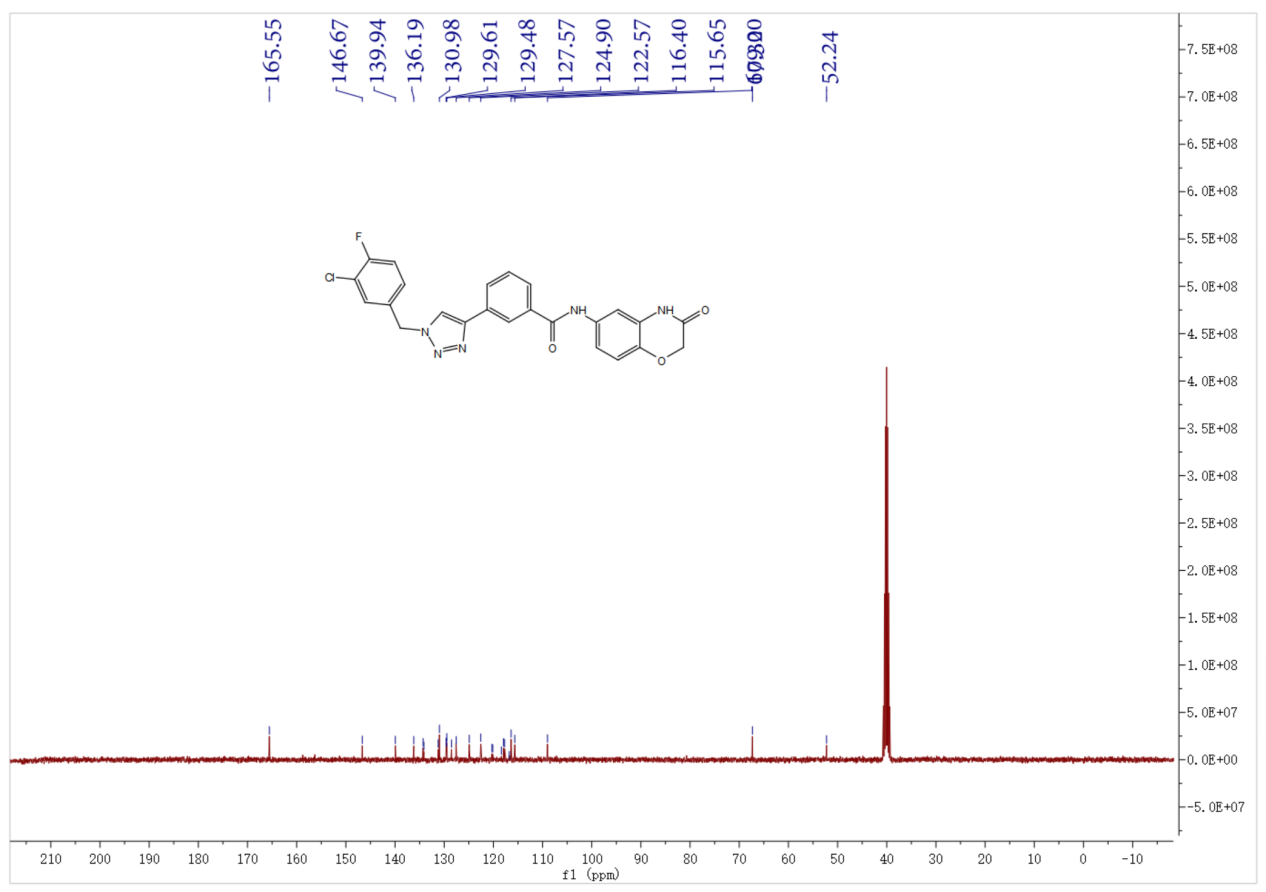


# Figure 8. ^1^H NMR and ^13^C NMR spectrums of compound 14g


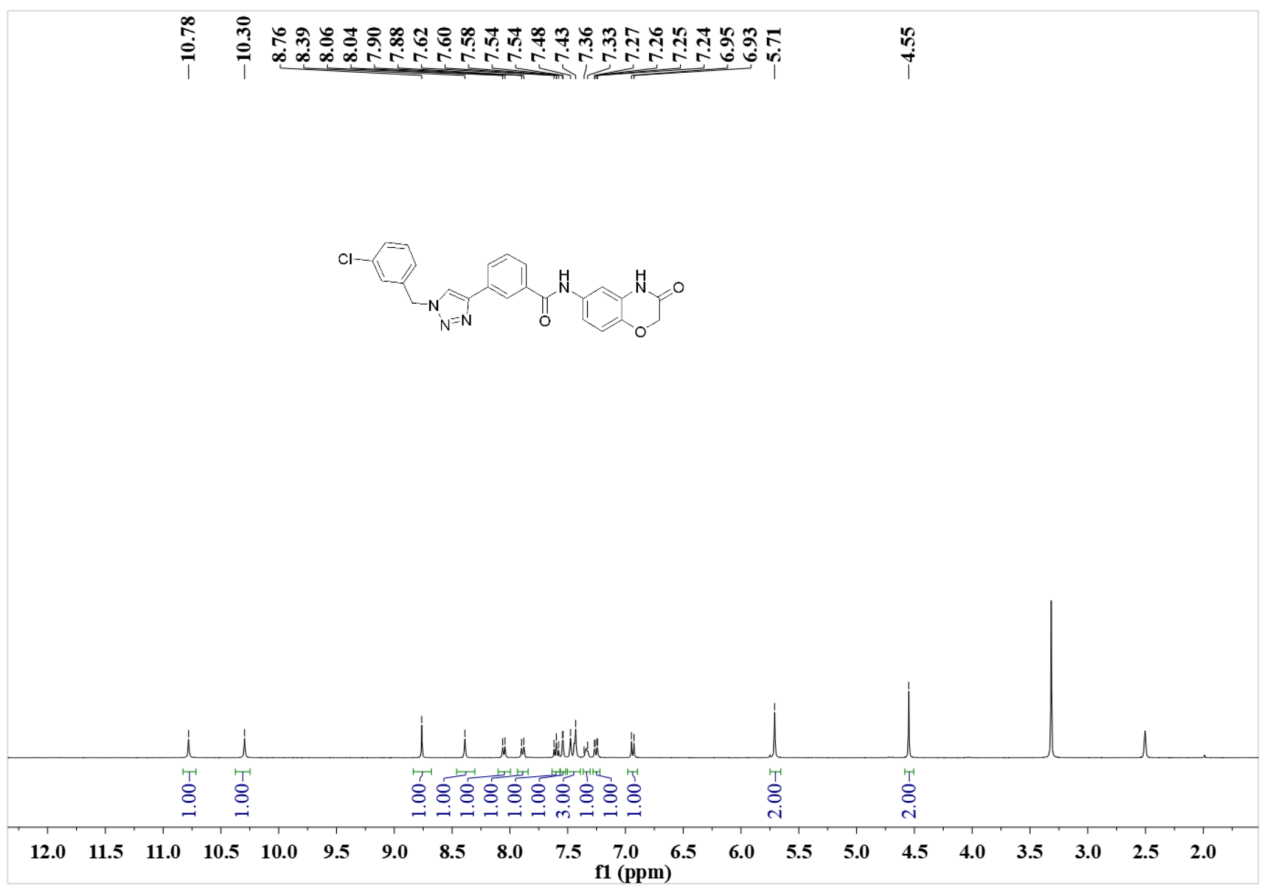


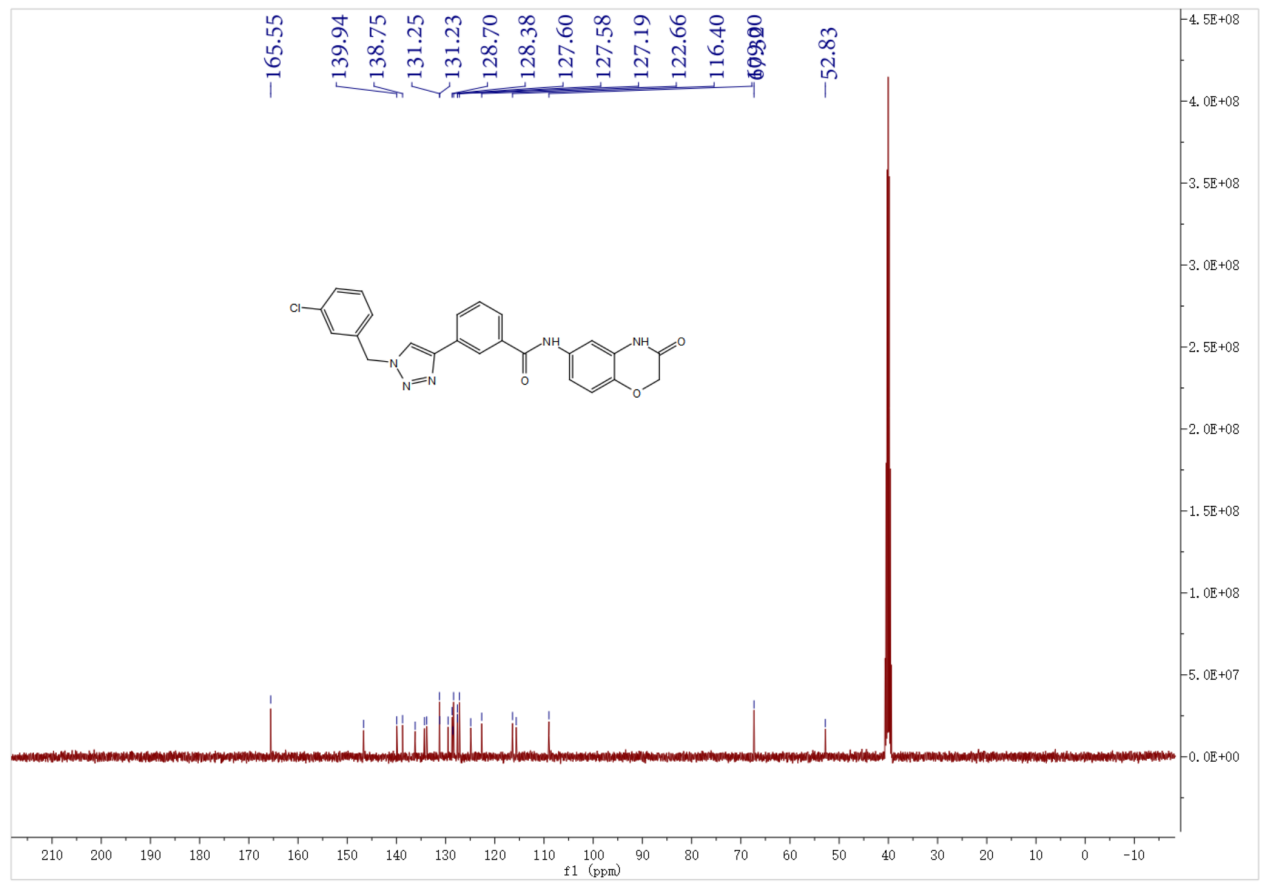


# Figure 9. ^1^H NMR and ^13^C NMR spectrums of compound 14h


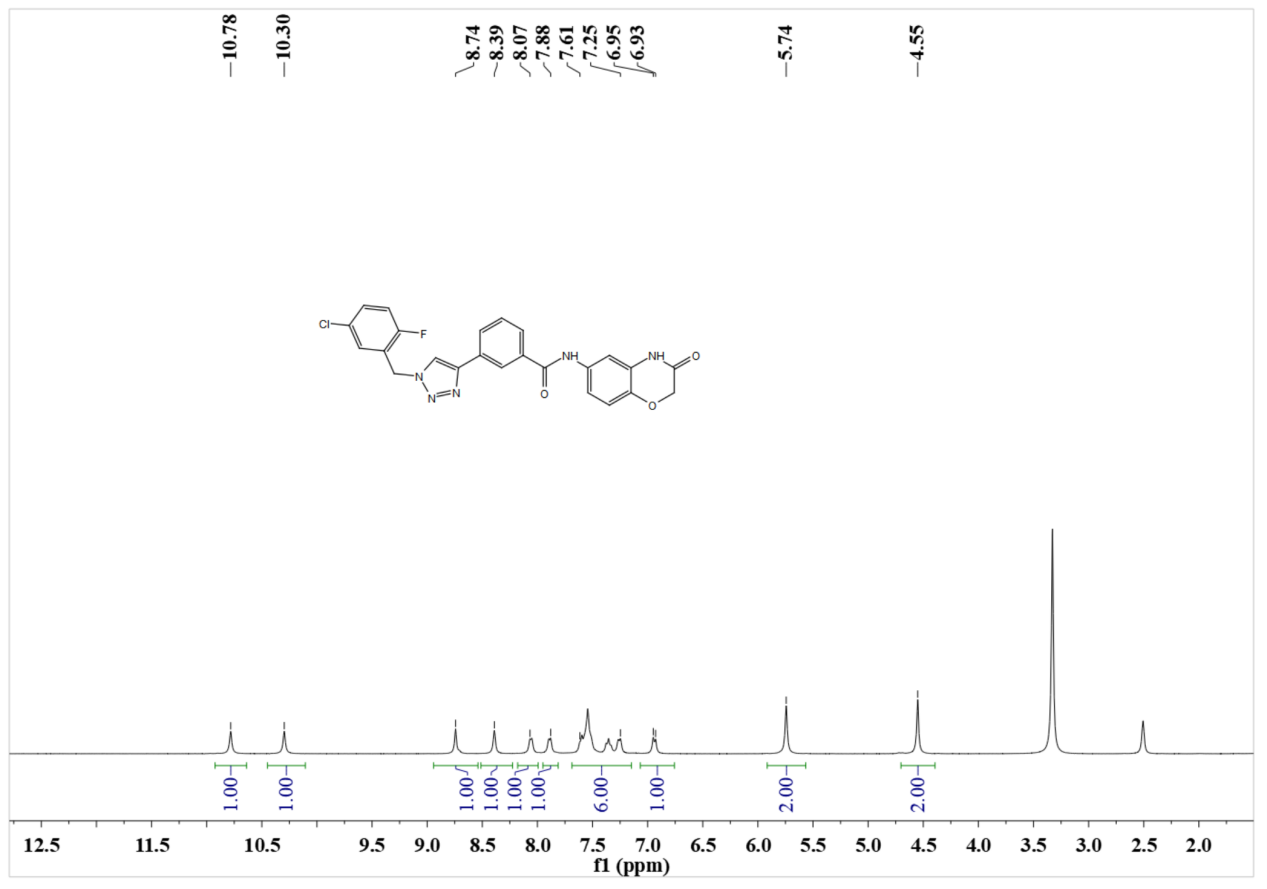


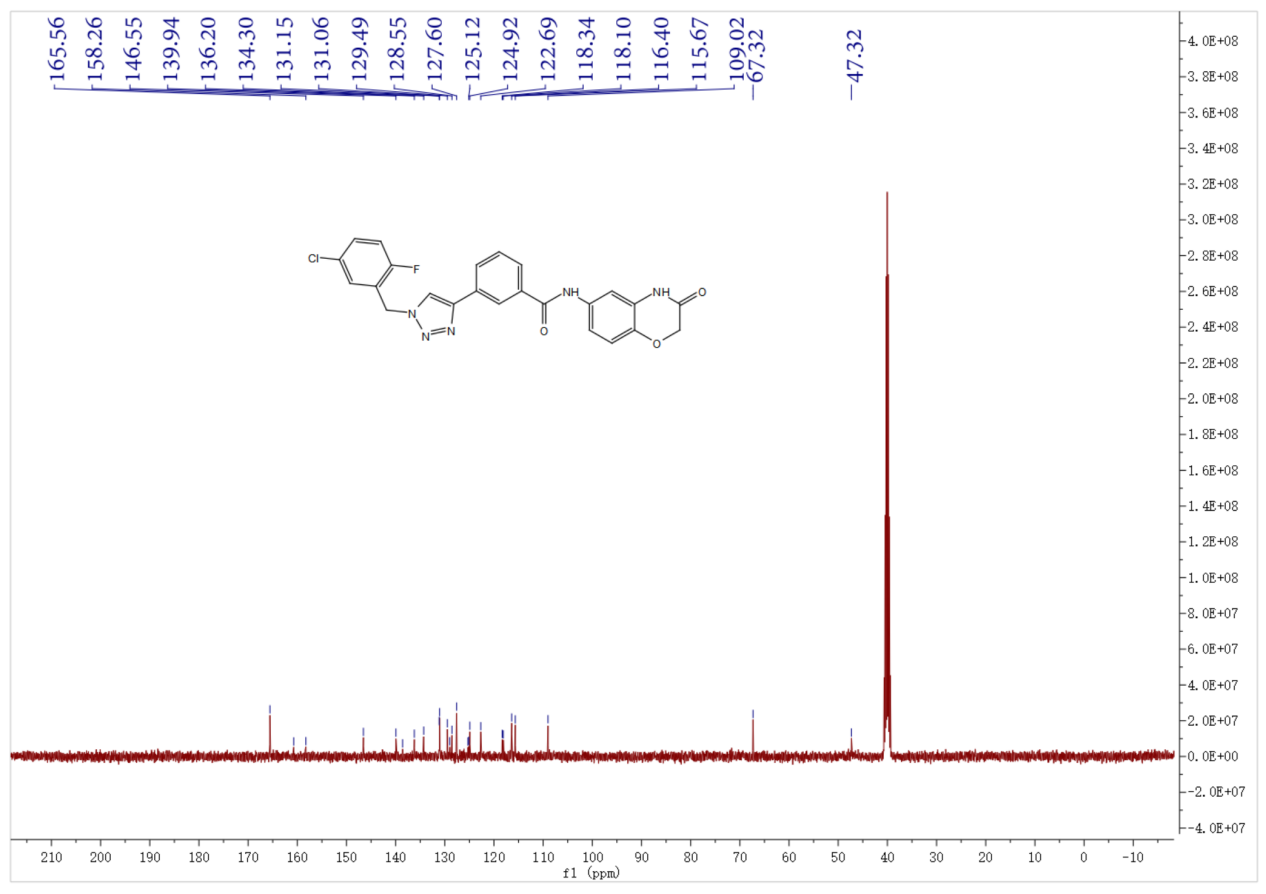


# Figure 10. ^1^H NMR and ^13^C NMR spectrums of compound 14i


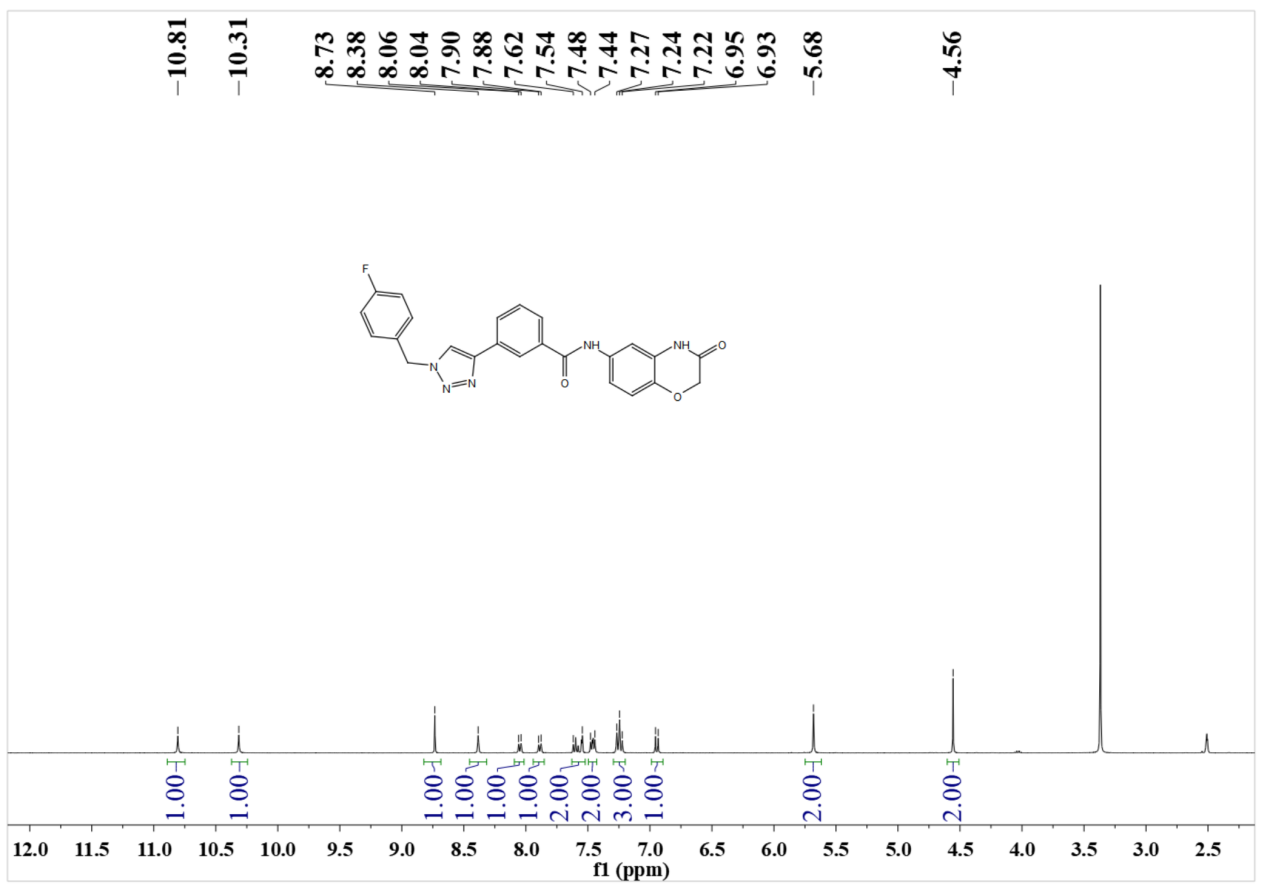


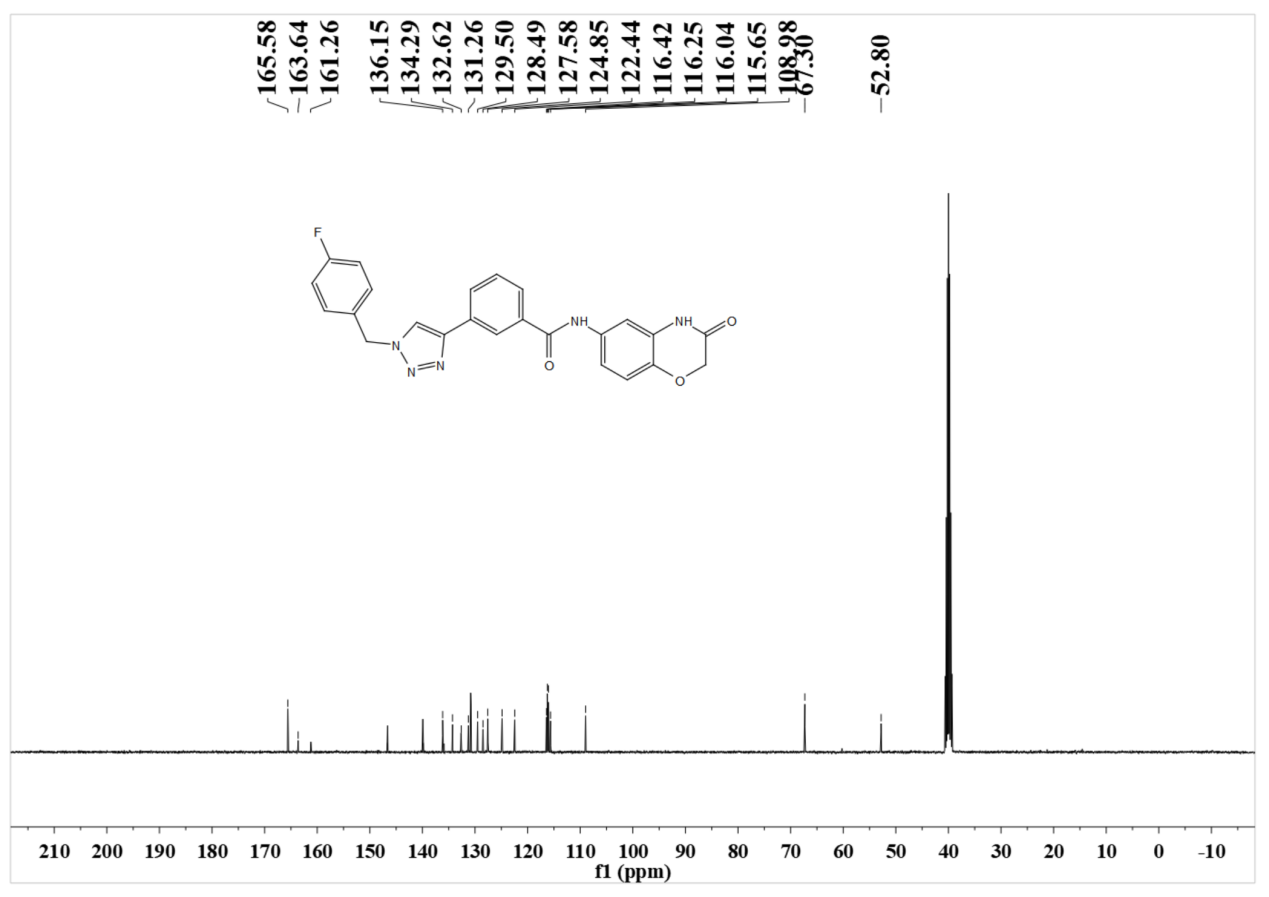


# Figure 11. ^1^H NMR and ^13^C NMR spectrums of compound 14j


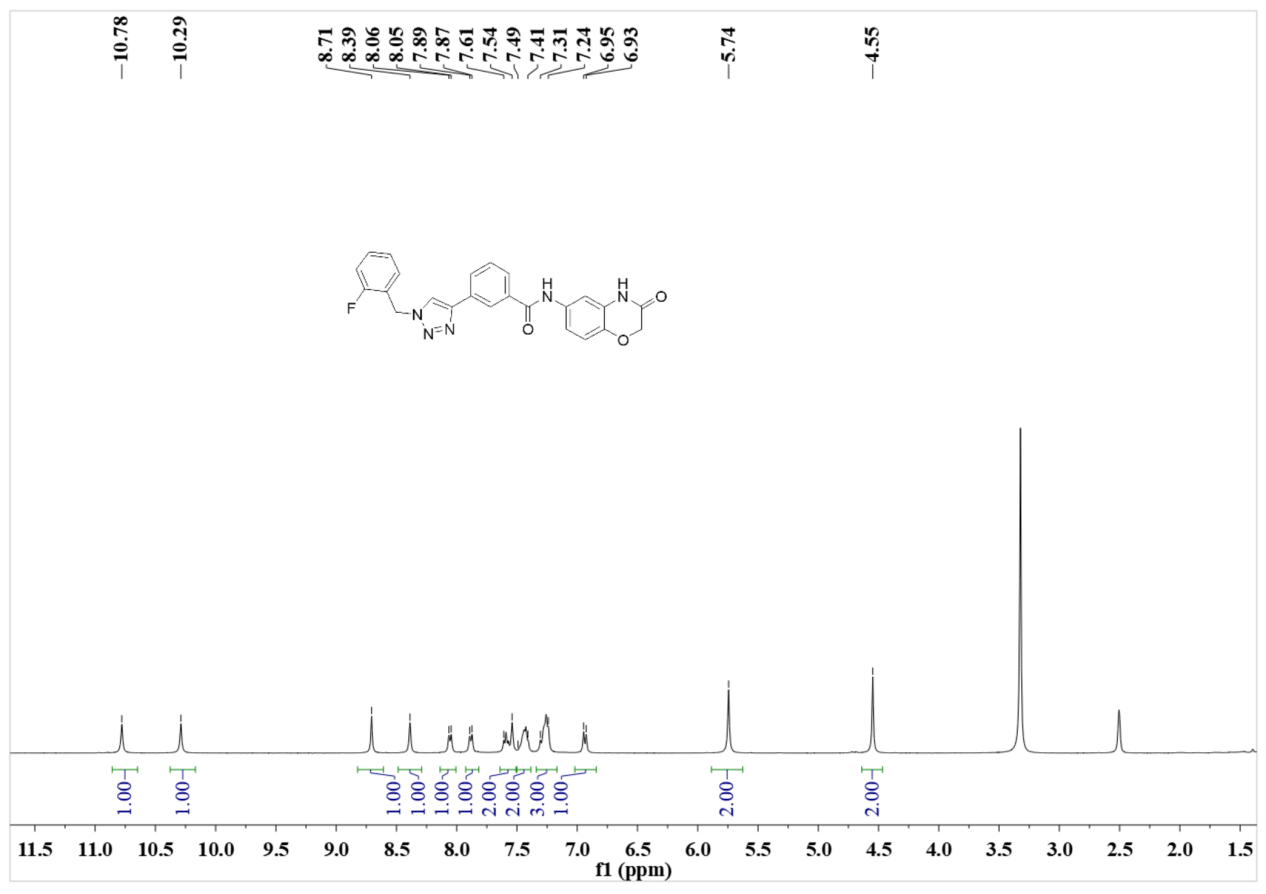


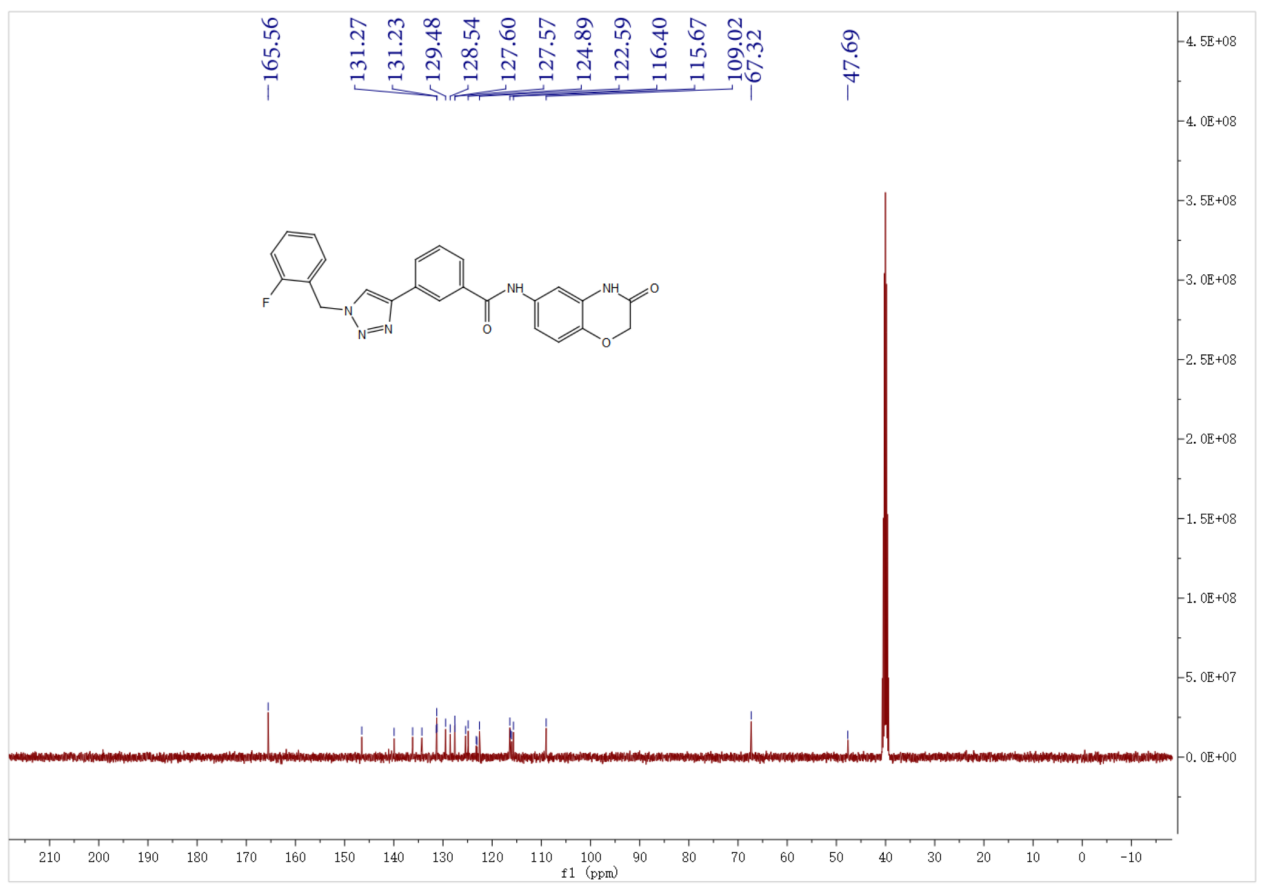

Supplement: Supplementary file 1 [file Supplementaryfile1.docx]
