# Supplementary figures and images for: Rational design of indoleamine 2,3-dioxygenase 1 (IDO1) inhibitors featuring 1,2,3-triazole derivatives with enhanced anti-inflammatory and analgesic efficacy
Source: Front Pharmacol. 2025 Sep 19;16:1574007. doi: 10.3389/fphar.2025.1574007 (PMC12491243; doi:10.3389/fphar.2025.1574007)

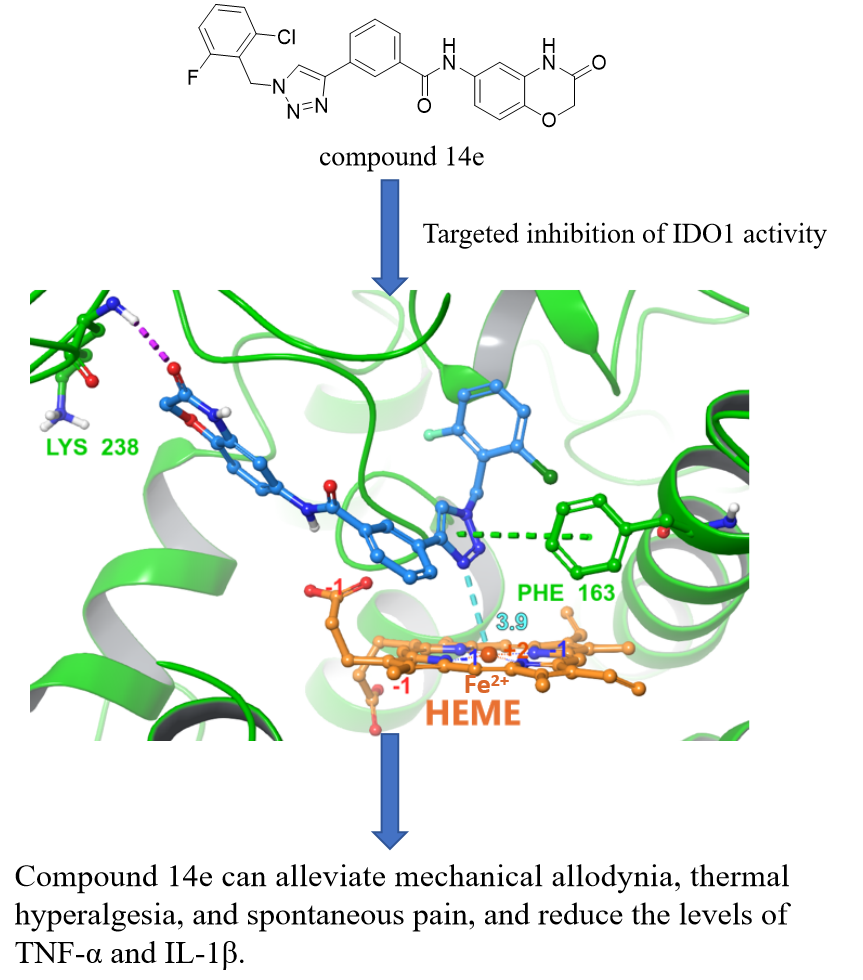

Supplement: Supplementary file 2 [file Supplementaryfile2.docx]
